# Supplementary material for: Actin Mutations and Their Role in Disease
Source: Int J Mol Sci. 2020 May 10;21(9):3371. doi: 10.3390/ijms21093371 (PMC7247010; doi:10.3390/ijms21093371)
Supplement: Supplementary file 1 [file ijms-21-03371-s001.pdf]

Supplemental Data:

Supplemental Table 1: Mutations in the actin isoforms.
1a: Mutations in $\alpha$ actin (skeletal, cardiac and smooth)

| Amino Acid Mutation* | Gene | Phenotype | SD | Ref. |
|---|---|---|---|---|
| Asp1Tyr | *ACTA1* | Core myopathy | SD1 | [1] |
| Asp1His | *ACTC1* | DCM | | [2] |
| Glu3Gln | *ACTA2* | Thoracis aortic disorder | | [3] |
| Glu4Lys | *ACTA1* | Nemaline myopathy | | [4] |
| Leu8Met | *ACTC1* | HCM | | [5] |
| Asp11Asn | *ACTA1* | Nemaline myopathy | | [4] |
| Gly15Arg | *ACTA1* | Actin myopathy | | [6] |
| Gly15Ser | *ACTA1* | Nemaline Myopathy | | [7] |
| Gly15Asp | *ACTA1* | Fetal akinesia | | [8] |
| Cys17Arg | *ACTA2* | Aortic disease | | [9] |
| Ala19Val | *ACTC1* | HCM, myocardial noncompaction & transmural crypts | | [10] |
| Gly20Ser | *ACTA2* | Aortic disease | | [9] |
| Phe21Leu | *ACTC1* | HCM | | [11] |
| Asp24Tyr | *ACTA2* | Thoracic aortic aneurysms and dissections | | [12] |
| Asp25Asn | *ACTC1* | HCM | | [2] |
| Asp25Asn | *ACTA1* | Nemaline Myopathy | | [13] |
| Asp25Gly | *ACTA2* | Thoracic aortic aneurysms and dissections | | [14] |
| Ala26Val | *ACTC1* | HCM | | [2] |
| Val35Leu | *ACTA1* | Nemaline Myopathy | SD2 | [13] [4] |
| Val35Ala | *ACTA1* | Nemaline myopathy | | [4] |
| Val35Ala | *ACTC1* | HCM | | [2] |
| Gly36Ala | *ACTA1* | Nemaline myopathy | | [4] |
| Gly36Arg | *ACTA2* | Thoracic aortic aneurysms and dissections | | [15] |
| Arg37Cys | *ACTA2* | Thoracic aortic aneurysms and dissections | | [16] |
| Arg37Gly | *ACTA2* | Aortic disease | | [9] |
| Arg38His | *ACTA1* | Fetal abnormalities | | [17] |
| Arg38His | *ACTA2* | Thoracic aortic disease, coronary artery disease and strokes | | [18] |
| Arg37Ser | *ACTA2* | Aortic dissection, acute | | [19] |
| Pro38Leu | *ACTA1* | Nemaline myopathy | | [13] |
| Pro38Ser | *ACTA2* | Thoracic aortic aneurysms and dissections | | [20] |
| Arg39Term | *ACTA1* | Nemaline myopathy | | [13] |
| His40Asn | *ACTA2* | Thoracic aortic aneurysms and dissections | | [15] |
| His40Tyr | *ACTA1* | Nemaline myopathy | | [6] |
| His40Tyr | *ACTC1* | HCM | | [2] |
| Gln41Arg | *ACTA1* | Nemaline myopathy | | [21] |
| Gly42Val | *ACTA1* | Nemaline myopathy | | [13, 22] |
| Val43Leu | *ACTA2* | Thoracic aortic aneurysms and dissections | | [23] |
| Val43Phe | *ACTA1* | Nemaline myopathy | | [13] |
| Met44Arg | *ACTA2* | Patent Ductus Arteriosus | | [24] |
| Met44Thr | *ACTA1* | Nemaline myopathy | | [4] |
| Gly46Ala | *ACTA1* | Fibre type disproportion, congenital and DCM | | [25] |
| Gly46Asp | *ACTA1* | Nemaline myopathy | | [4] |
| Gly46Cys | *ACTA1* | Nemaline myopathy | | [4] |
| Gly46Ser | *ACTA1* | Congenital myopathy, mild | | [26] |

| | | | | |
|---|---|---|---|---|
| Met47Val | *ACTA1* | Nemaline myopathy | | [4] |
| Met47Val | *ACTA2* | Thoracic aortic aneurysms and dissections | | [16] |
| Met47Leu | *ACTC1* | HCM | | [22] |
| Gly48Asp | *ACTA1* | Prominent finger flexor and rimmed vacuoles | | [27] |
| Gly48Cys | *ACTA1* | Congenital myopathy with fibre type disproportion | | [28] |
| Gly48Val | *ACTA2* | Aortic disease | | [9] |
| Gly48Ser | *ACTC1* | HCM | | [29] |
| Gly55Arg | *ACTA1* | Nemaline myopathy | | [4] |
| Asp56Asn | *ACTA2* | Aortic dissection, acute | | [19] |
| Ala58Glu | *ACTA2* | Thoracic aortic disorder | | [3] |
| Gln59Arg | *ACTA2* | Thoracic aortic aneurysms and dissections | | [15] |
| Arg62Lys | *ACTA2* | Aortic disease | | [9] |
| Gly63Asp | *ACTC1* | HCM | | [30] |
| Ile64Asn | *ACTA1* | Nemaline myopathy | | [13] |
| Ile64Ser | *ACTA1* | Nemaline myopathy | | [4] |
| Thr66Asn | *ACTA1* | Nemaline myopathy | | [4] |
| Thr66Ile | *ACTA1* | Nemaline myopathy | | [31] |
| Leu67Gln | *ACTA2* | Aortic disease | | [9] |
| Lys68Arg | *ACTA1* | Nemaline myopathy | | [4] |
| Pro70Arg | *ACTA1* | Congenital myopathy | | [32] |
| Pro70Gln | *ACTA2* | Thoracic aortic aneurysms and dissections | | [18] |
| Ile71Phe | *ACTA1* | Nemaline myopathy | | [4] |
| Ile71Val | *ACTA1* | Nemaline myopathy | | [4] |
| Glu72Lys | *ACTA1* | Nemaline myopathy | | [31] |
| His73Arg | *ACTA1* | Nemaline myopathy | | [13] |
| His73Asn | *ACTA1* | Nemaline myopathy | | [4] |
| His73Leu | *ACTA1* | Nemaline myopathy | | [13] |
| Gly74Asp | *ACTA1* | Nemaline myopathy | | [33] |
| Ile75Leu | *ACTA1* | Nemaline myopathy | | [13] |
| Ile75Ser | *ACTA1* | Nemaline myopathy | | [4] |
| Ile75Val | *ACTC1* | HCM | | [2] |
| Ile76Asn | *ACTC1* | Developmental disorder | | [34] |
| The77Ala | *ACTA1* | Nemaline myopathy | | [13] |
| Asp80Glu | *ACTA2* | Thoracic aortic aneurysms and dissections | | [23] |
| Met82Thr | *ACTC1* | Congenital heart defects | | [35] |
| Glu83Lys | *ACTA1* | Nemaline myopathy | | [36] |
| Trp86Arg | *ACTA2* | Aortic disease | | [9] |
| His88Tyr | *ACTC1* | HCM | SD1 | [37] |
| Tyr91Cys | *ACTC1* | HCM | | [38] |
| Tyr91His | *ACTC1* | Noncompaction, left ventricular | | [39] |
| Asn92Lys | *ACTA1* | Congenital myopathy, nemaline myopathy | | [40], [4] |
| Asn92Ser | *ACTC1* | Noncompaction, left ventricular | | [41] |
| Leu94Pro | *ACTA1* | Nemaline myopathy | | [6] |
| Arg95Cys | *ACTC1* | HCM | | [37] |
| Glu99Lys | *ACTC1* | HCM | | [42] |
| His101Gln | *ACTC1* | HCM | | [30] |
| Thr106Met | *ACTA2* | Thoracic aortic aneurysm | | [43] |
| Glu107Asp | *ACTA1* | Nemaline myopathy | | [4] |
| Ala108Val | *ACTC1* | Noncompaction, left ventricular | | [44] |
| Asn111Ser | *ACTA1* | Nemaline myopathy | | [45] |
| Asn111Thr | *ACTA2* | Thoracic aortic aneurysms and dissections | | [46] |
| Pro112Leu | *ACTA1* | Muscular dystrophy | | [47] |
| Lys113Glu | *ACTA1* | Nemaline myopathy | | [4] |

| | | | | |
|---|---|---|---|---|
| Ala114Ser | *ACTA1* | Nemaline myopathy | | [36] |
| Ala114Thr | *ACTA1* | Nemaline myopathy | | [13] |
| Ala114Val | *ACTA1* | Congenital myopathy | | [32] |
| Asn115Ile | *ACTA2* | Thoracic aortic aneurysms and dissections | | [48] |
| Asn115Ser | *ACTA1* | Nemaline myopathy | | [6] |
| Asn115Ser | *ACTA2* | Thoracic aortic aneurysms and dissections | | [14] |
| Asn115Thr | *ACTA1* | Nemaline myopathy | | [13] |
| Asn115Thr | *ACTA2* | Thoracic aortic aneurysms and dissections | | [49] |
| Arg116Gln | *ACTA2* | Thoracic aortic aneurysms and dissections | | [49] |
| Arg116His | *ACTA1* | Nemaline myopathy | | [13] |
| Glu117Gln | *ACTA1* | Nemaline myopathy | | [50] |
| Glu117Gln | *ACTC1* | DCM | | [51] |
| Met119Val | *ACTC1* | HCM | | [52] |
| Thr120Ser | *ACTA1* | Nemaline myopathy | | [4] |
| Met123Val | *ACTC1* | Atrial septal defect | | [53] |
| Thr126Il2 | *ACTC1* | DCM | | [54] |
| Met132Ile | *ACTA1* | Nemaline myopathy | | [4] |
| Met132Thr | *ACTA2* | Aortic dissection | | [55] |
| Met132Val | *ACTA1* | Nemaline myopathy | | [6] |
| Tyr133His | *ACTA2* | Thoracic aortic aneurysms and dissections | | [49] |
| Val134Ala | *ACTA1* | Nemaline myopathy | | [13] |
| Ile136Met | *ACTA1* | Nemaline myopathy | | [56] |
| Ile136Thr | *ACTA1* | Nemaline myopathy | | [4] |
| Gln137His | *ACTA1* | Nemaline myopathy | | [57] |
| Ala138Asp | *ACTA1* | Nemaline myopathy | | [4] |
| Ala138Pro | *ACTA1* | Nemaline myopathy | | [13] |
| Ala138Val | *ACTA2* | Thoracic aortic aneurysms and dissections | | [46] |
| Val139Ala | *ACTA1* | Nemaline myopathy | | [4] |
| Val139Ala | *ACTA2* | Aortic disease | | [9] |
| Leu140Pro | *ACTA1* | Nemaline myopathy | | [13] |
| Leu142Phe | *ACTA1* | Nemaline myopathy | | [58] |
| Tyr143Cys | *ACTA2* | Thoracic aortic aneurysms and dissections | | [59] |
| Tyr143Term | *ACTA1* | Nemaline myopathy | | [4] |
| Ala144Val | *ACTA1* | Muscular dystrophy, limb girdle | | [60] |
| Gly146Arg | *ACTA2* | Aortic disease | | [9] |
| Gly146Asp | *ACTA1* | Actin myopathy | | [13] |
| Gly146Ser | *ACTA1* | Nemaline myopathy | | [4] |
| Arg147Cys | *ACTA2* | Thoracic aortic aneurysms and dissections | | [49] |
| Arg147Lys | *ACTA1* | Nemaline myopathy | | [4] |
| Thr148Ala | *ACTA1* | Nemaline myopathy | | [61] |
| Thr148Asp | *ACTA1* | Nemaline myopathy | | [13] |
| Thr148Ile | *ACTA1* | Intranuclear rod myopathy | | [62] |
| Thr148Ser | *ACTA1* | Nemaline myopathy | | [4] |
| Gly150Ala | *ACTA1* | Congenital myopathy | | [63] |
| Val152Ala | *ACTA2* | Thoracic aortic aneurysms and dissections | SD3 | [49] |
| Val152Leu | *ACTA1* | Muscular dystrophy with rigid spine | | [64] |
| Asp154Asn | *ACTA1* | Actin myopathy | | [13] |
| Asp157Asn | *ACTC1* | DCM | | [2] |
| Gly158Asp | *ACTA2* | Thoracic aortic aneurysms and dissections | | [18] |
| Gly158Cys | *ACTA1* | Nemaline myopathy | | [4] |
| Val159Ala | *ACTA2* | Aortic aneurysm | | [65] |
| His161Asp | *ACTA1* | Nemaline myopathy | | [4] |
| His161Gln | *ACTA2* | Aortic disease | | [9] |
| Val163Leu | *ACTA1* | Actin myopathy | | [13] |
| Val163Leu | *ACTA1* | Actin myopathy | | [6] |

| | | | | |
|---|---|---|---|---|
| Val163Met | *ACTA1* | Nemaline myopathy | | [13] |
| Pro164Ala | *ACTC1* | HCM | | [42] |
| Pro164Thr | *ACTA2* | Aortic disease | | [9] |
| Tyr166Asn | *ACTA2* | Thoracic aortic aneurysms and dissections | | [48] |
| Tyr166Cys | *ACTC1* | HCM | | [66] |
| Ala170Glu | *ACTA1* | Nemaline myopathy | | [4] |
| Ala170GLy | *ACTA1* | Nemaline myopathy | | [13] |
| Ala170Thr | *ACTC1* | HCM | | [11] |
| His173Arg | *ACTC1* | DCM | | [67] |
| Met176Ley | *ACTC1* | Atrial septal defect | | [68] |
| Arg177Cys | *ACTA2* | Cardiovascular, autonomic and brain anomalies | | [69] |
| Arg177His | *ACTA2* | Multisystem smooth muscle dysfunction | | [70] |
| Arg177Leu | *ACTA2* | Cerebrovascular disease | | [71] |
| Arg177Ser | *ACTA2* | Multisystem smooth muscle dysfunction | | [9] |
| Leu178Pro | *ACTA1* | Nemaline myopathy | | [4] |
| Asp179Asn | *ACTA1* | Nemaline myopathy | | [13] |
| Asp179Gly | *ACTA1* | Nemaline myopathy | | [36] |
| Asp179His | *ACTA1* | Nemaline myopathy | | [13] |
| Ala181Thr | *ACTA1* | Nemaline myopathy | | [4] |
| Gly182Asp | *ACTA1* | Nemaline myopathy | | [6] |
| Arg183Cys | *ACTA1* | Nemaline myopathy | | [6] |
| Arg183Gln | *ACTA2* | Thoracic disease and coronary artery disease | | [18] |
| Arg183Gly | *ACTA1* | Nemaline myopathy | | [56] |
| Arg183Leu | *ACTA1* | Nemaline myopathy | | [4] |
| Arg183Ser | *ACTA1* | Nemaline myopathy | | [13] |
| Asp184Gly | *ACTA1* | Nemaline myopathy | | [4] |
| Asp184His | *ACTA1* | Myopathy, thin filament | | [72] |
| Tyr188Ser | *ACTA2* | Marfan syndrome with aortopathy | | [73] |
| Tyr188Term | *ACTA1* | Nemaline myopathy | | [4] |
| Leu189Pro | *ACTA1* | Myopathy | | [74] |
| Met190Val | *ACTA2* | Aortic disease | | [55] |
| Lys191Asn | *ACTA1* | Nemaline myopathy | | [4] |
| Thr194Pro | *ACTA1* | Nemaline myopathy | | [4] |
| Glu195Asp | *ACTA1* | Distal myopathy with nemaline rods, neuromuscular disorder | | [75] |
| Arg196Cys | *ACTA1* | Nemaline myopathy | | [4] |
| Arg196Cys | *ACTA2* | Aortic disease | SD4 | [9] |
| Arg196His | *ACTA1* | Nemaline myopathy | | [4] |
| Arg196His | *ACTA2* | Aortic disease | | [9] |
| Arg196Leu | *ACTA1* | Nemaline myopathy | | [13] |
| Arg196Ser | *ACTA1* | Nemaline myopathy | | [4] |
| Gly197Ser | *ACTA1* | Nemaline myopathy | | [36] |
| Tyr198Cys | *ACTA1* | Nemaline myopathy | | [4] |
| Val201Ile | *ACTA2* | Thoracic aortic aneurysms and dissections | | [46] |
| Val201Leu | *ACTA2* | Thoracic aortic disorder | | [3] |
| Thr202Ile | *ACTA1* | Nemaline myopathy | | [76] |
| Ala204Thr | *ACTA1* | Nemaline myopathy | | [4] |
| Glu205Asp | *ACTA1* | Congenital myopathy with fibre-type disproportion | | [77] |
| Glu205Gly | *ACTA1* | Nemaline myopathy | | [4] |
| Arg206His | *ACTC1* | DCM | | [78] |
| Glu207Asp | *ACTA1* | Nemaline myopathy | | [79] |
| Arg210Gln | *ACTA2* | Thoracic aortic disease, coronary artery disease and strokes | | [18] |
| Arg210His | *ACTC1* | Noncompaction, left ventricular, DCM, HCM | | [80] |

| | | | | |
|---|---|---|---|---|
| Lys215Term | *ACTA1* | Nemaline myopathy | | [4] |
| Tyr218His | *ACTC1* | DCM | | [67] |
| Tyr218Ser | *ACTC1* | Noncompaction, left ventricular | | [81] |
| Leu221Pro | *ACTA1* | Congenital myopathy with fibre-type disproportion | | [4] |
| Asp222Tyr | *ACTC1* | Noncompaction, left ventricular | | [41] |
| Glu224Gln | *ACTA1* | Nemaline myopathy | | [13] |
| Glu224Gly | *ACTA1* | Nemaline myopathy | | [13] |
| Glu226Gln | *ACTA1* | Nemaline myopathy | | [4] |
| Glu226Term | *ACTA1* | Myopathy, early onset | | [82] |
| Met227Ile | *ACTA1* | Nemaline myopathy | | [13] [4] |
| Met227Thr | *ACTA1* | Nemaline myopathy | | [13] |
| Met227Val | *ACTA1* | Nemaline myopathy | | [13] |
| Thr229Arg | *ACTC1* | Noncompaction, left ventricular with arrhythmias | | [83] |
| Ala230Thr | *ACTC1* | HCM and arrhythmias | | [84] |
| Ala230Val | *ACTC1* | HCM | | [85] |
| Ala230Val | *ACTA1* | Nemaline myopathy | | [4] |
| Ser234Phe | *ACTC1* | HCM | | [86] |
| Glu237Lys | *ACTA1* | Nemaline myopathy and HCM | | [87] |
| Glu237Term | *ACTA1* | Nemaline myopathy | | [4] |
| Glu241Lys | *ACTA1* | Nemaline myopathy | | [13] |
| Glu241Lys | *ACTA2* | Thoracic aortic aneurysms and dissections | | [23] |
| Leu242Phe | *ACTA2* | Aortic disease | | [9] |
| Pro243His | *ACTA2* | Thoracic aortic disease and strokes | | [18] |
| Pro243Leu | *ACTA2* | Aortic disease | | [9] |
| Asp244Glu | *ACTA1* | Nemaline myopathy | | [4] |
| Gly245Arg | *ACTA1* | Nemaline myopathy | | [4] |
| Gln246Arg | *ACTA1* | Nemaline myopathy | | [13] |
| Gln246Lys | *ACTA1* | Nemaline myopathy | | [13] |
| Ile248Leu | *ACTA2* | Thoracic aortic disease and strokes | | [18] |
| Ile248Thr | *ACTA1* | Myofibrillar myopathy | | [88] |
| Thr249Ser | *ACTC1* | HCM | | [2] |
| Ile250Met | *ACTC1* | DCM | | [54] |
| Ile250Thr | *ACTC1* | DCM | | [78] |
| Gly251Arg | *ACTA1* | Distal myopathy | | [89] |
| Gly251Asp | *ACTA1* | Nemaline myopathy | | [13] |
| Asn252Tyr | *ACTA1* | Nemaline myopathy | | [4] |
| Glu253Gly | *ACTA1* | Nemaline myopathy | | [4] |
| Arg254Gly | *ACTA1* | DCM, skeletal myopathy | | [90] |
| Arg254His | *ACTA1* | DCM | | [90] |
| Arg254His | *ACTA2* | Thoracic aortic aneurysms and dissections | | [91] |
| Phe255Cys | *ACTA1* | Nemaline myopathy | | [4] |
| Arg256Cys | *ACTA2* | Thoracic aortic aneurysms and dissections | | [49] |
| Arg256His | *ACTA1* | Nemaline myopathy | | [6] |
| Arg256His | *ACTA2* | Thoracic aortic disease and strokes | | [18] |
| Arg256Leu | *ACTA1* | Nemaline myopathy | | [13] |
| Glu259Val | *ACTA1* | Nemaline myopathy | | [6] |
| Gln263Glu | *ACTC1* | HCM | | [2] |
| Gln263Leu | *ACTA1* | Nemaline myopathy | | [6] |
| Pro264Leu | *ACTC1* | HCM | | [2] |
| Pro264Thr | *ACTA1* | Nemaline myopathy | | [4] |
| Ser265Cys | *ACTA1* | Nemaline myopathy | | [4] |
| Phe266Leu | *ACTA1* | Nemaline myopathy | | [92] |
| Ile267Thr | *ACTC1* | DCM | | [93] |

| | | | | |
|---|---|---|---|---|
| Gly268Arg | *ACTA1* | Nemaline myopathy | | [13, 94] |
| Gly268Arg | *ACTA2* | Aortic disease | | [9] |
| Gly268Asp | *ACTA1* | Nemaline myopathy | | [94] |
| Gly268Cys | *ACTA1* | Nemaline myopathy | | [56] |
| Gly268Glu | *ACTA2* | Thoracic aortic aneurysm | | [43] |
| Gly268Ser | *ACTA1* | Nemaline myopathy | | [79] |
| Met269Arg | *ACTA1* | Nemaline myopathy | | [95] |
| Met269Val | *ACTA1* | Facioscapuloperoneal myopathy | | [96] |
| Met269Val | *ACTC1* | Cardiomyopathy, noncompaction, left ventricular | | [97] |
| Glu270Gln | *ACTA1* | Nemaline myopathy | | [4] |
| Ser271Phe | *ACTC1* | HCM | | [98] |
| Gly273Ala | *ACTA2* | Thoracic aortic aneurysms and dissections | | [9] |
| Ala282Glu | *ACTA1* | Nemaline myopathy | | [13] |
| Ala272Val | *ACTA1* | Myopathy | | [74] |
| Thr277Ala | *ACTA2* | Thoracic aortic disorder | | [3] |
| Tyr279His | *ACTA1* | Nemaline myopathy | | [13] |
| Asn280Lys | *ACTA1* | Nemaline myopathy | | [6] |
| Ile282Asn | *ACTA2* | Thoracic aortic aneurysms and dissections | | [91] |
| Ile282Phe | *ACTC1* | HCM | | [2] |
| Met283Arg | *ACTA1* | Nemaline myopathy | | [4] |
| Met283Lys | *ACTA1* | Nemaline myopathy | | [13] |
| Met283Thr | *ACTA2* | Thoracic aortic disorder | | [3] |
| Asp286Gly | *ACTA1* | Nemaline myopathy | | [6] |
| Ile287Thr | *ACTC1* | Cardiomyopathy, noncompaction, left ventricular | | [99] |
| Asp288Asn | *ACTA1* | Nemaline myopathy | | [4] |
| Asp288His | *ACTA1* | Nemaline myopathy | | [100] |
| Ile289Phe | *ACTA1* | Nemaline myopathy | | [4] |
| Arg290Gly | *ACTC1* | Thoracic aortic aneurysms and dissections | | [49] |
| Asp292Val | *ACTA1* | Congenital myopathy with fibre-type disproportion | | [4] |
| Tyr294Asn | *ACTC1* | HCM | SD3 | [101] |
| Tyr294His | *ACTC1* | HCM, DCM, noncompaction left ventricular | | [102] |
| Ala295Ser | *ACTC1* | HCM | | [103] |
| Ala295Thr | *ACTA1* | Myopathy/muscular dystrophy | | [104] |
| Met299Lys | *ACTA1* | Nemaline myopathy | | [4] |
| Ser300Ala | *ACTA2* | Thoracic aortic aneurysms and dissections | | [9] |
| Gly302Arg | *ACTA2* | Thoracic aortic aneurysms and dissections | | [16] |
| Gly302Ser | *ACTA2* | Thoracic aortic disorder, nonsyndromic | | [105] |
| Met305Leu | *ACTC1* | HCM | | [66] |
| Tyr306Cys | *ACTA1* | Muscular dystrophy and congenital myopathy | | [106] |
| Asp311His | *ACTC1* | Cardiomyopathy, restrictive | | [107] |
| Arg312Cys | *ACTC1* | HCM | | [108] |
| Arg312His | *ACTC1* | DCM | | [109] |
| Arg312Term | *ACTA2* | Thoracic aortic aneurysms and dissections | | [15] |
| Ala321Val | *ACTC1* | HCM | | [110] |
| Ser323Arg | *ACTA1* | Muscle weakness | | [111] |
| Thr324Asn | *ACTA2* | Thoracic aortic disease, coronary artery disease and strokes | | [18] |
| Met325Lys | *ACTA1* | Fibre-type disproportion, congenital | | [112] |
| Lys326Asn | *ACTA1* | Nemaline myopathy | | [4] |
| Lys326Asn | *ACTA2* | Thoracic aortic aneurysms and dissections | | [113] |

| | | | | |
|---|---|---|---|---|
| Ile327Thr | *ACTC1* | Cardiomyopathy, non-compaction, left ventricular | | [114] |
| Ile329Asn | *ACTC1* | HCM | | [86] |
| Ala331Pro | *ACTC1* | HCM | | [86] |
| Pro332Arg | *ACTA1* | Nemaline myopathy | | [4] |
| Pro332Ser | *ACTA1* | Congenital myopathy with fibre-type disproportion | | [115] |
| Glu334Ala | *ACTA1* | Core myopathy | | [1] |
| Glu334Lys | *ACTA1* | Nemaline myopathy | | [4] |
| Lys336GLu | *ACTA1* | Nemaline myopathy, HCM | | [116] |
| Lys336Ile | *ACTA1* | Nemaline Myopathy | | [13] |
| Lys336Thr | *ACTA1* | Nemaline myopathy | | [117] |
| Gly342Ser | *ACTA2* | Aortic dissection, acute | | [19] |
| Leu346Arg | *ACTA2* | Thoracic aortic aneurysms and dissections | | [48] |
| Leu346Gln | *ACTA1* | Zebra-body myopathy | | [118] |
| Ser348Leu | *ACTA1* | Actin myopathy | | [13] |
| Thr351Ala | *ACTA1* | Myopathy/muscular dystrophy | | [104] |
| Thr351Asn | *ACTA2* | Thoracic aortic aneurysms and dissections | | [49] |
| Phe352Ser | *ACTA1* | Nemaline myopathy | | [4] |
| Phe352Tyr | *ACTA1* | Nemaline myopathy | | [4] |
| Met355Val | *ACTC1* | HCM | | [2] |
| Trp356Cys | *ACTA1* | Nemaline myopathy and DCM | | [119] |
| Ile357Leu | *ACTA1* | Nemaline myopathy | | [56] |
| Glu361Gly | *ACTC1* | DCM | | [109] |
| Ala365Thr | *ACTC1* | HCM | | [2] |
| Gly366Arg | *ACTA2* | Schizophrenia | | [120] |
| Pro367Leu | *ACTA1* | Nemaline myopathy | | [4] |
| Ile369Leu | *ACTA1* | Nemaline myopathy | SD1 | [92] |
| Ile369Phe | *ACTA1* | Nemaline myopathy | | [4] |
| Ile369Thr | *ACTC1* | DCM | | [121] |
| Val370Phe | *ACTA1* | Nemaline myopathy | | [6] |
| Arg372Cys | *ACTA1* | Nemaline myopathy | | [4] |
| Arg372Cys | *ACTA2* | Thoracic aortic disorder | | [3] |
| Arg372Ser | *ACTA1* | Nemaline myopathy | | [13] |
| Lys373Asn | *ACTA1* | Nemaline myopathy | | [4] |
| Lys373Gln | *ACTA1* | Nemaline myopathy | | [21] |
| Lys373Glu | *ACTA1* | Nemaline myopathy | | [94] |
| Cys374Ser | *ACTA1* | Nemaline myopathy | | [4] |
| Phe375Cys | *ACTA1* | Nemaline myopathy | | [122] |
| Phe375Tyr | *ACTA1* | Nemaline myopathy | | [4] |
| Term376Gln | *ACTA1* | Nemaline myopathy | | [123] |
| Term376Trp | *ACTA1* | Nemaline myopathy | | [123] |
| Term376Tyr | *ACTA1* | Nemaline myopathy | | [63] |

1b: Mutations in β and γ actin

| Amino Acid Mutation* | Gene | Phenotype | SD | Ref. |
|---|---|---|---|---|
| Asn10Asp | *ACTG1* | Baraitser-Winter syndrome | | [124] |
| Asn10Asp | *ACTB* | Baraitser-Winter syndrome | | [125] |
| Asn11His | *ACTB* | Baraitser-Winter syndrome | SD1 | [126] |
| Pro30Ser | *ACTG1* | Hearing loss, non-syndromic | | [127] |
| His38Tyr | *ACTG1* | Baraitser-Winter syndrome | | [128] |
| Val41Met | *ACTB* | Baraitser-Winter syndrome | | [129] |
| Met45Thr | *ACTB* | Baraitser-Winter syndrome | | [129] |
| Gly46Arg | *ACTG1* | Deafness, dominant progressive | | [130] |
| Asp49Asn | *ACTG1* | Deafness, dominant progressive | | [131] |
| Ala56Val | *ACTG1* | Baraitser-Winter syndrome | | [132] |
| Gln57Arg | *ACTB* | Baraitser-Winter syndrome | SD2 | [126] |
| Leu63Phe | *ACTB* | Baraitser-Winter syndrome | | [126] |
| Leu63Val | *ACTB* | Baraitser-Winter syndrome | | [125] |
| Thr64Ile | *ACTG1* | Hearing loss | | [133] |
| *Pro68Ala* | *ACTB* | Baraitser-Winter syndrome | | [134] |
| *Pro68Leu* | *ACTB* | Baraitser-Winter syndrome | | [126] |
| *Pro68Leu* | *ACTG1* | Ocular coloboma | | [135] |
| *His71Leu* | *ACTB* | Baraitser-Winter syndrome | | [136] |
| *Gly72Ser* | *ACTB* | Baraitser-Winter syndrome | | [137] |
| *Ile73Thr* | *ACTB* | Baraitser-Winter syndrome | | [126] |
| *Ile73Leu* | *ACTG1* | Microlissencephaly | | [138] |
| Met80Leu | *ACTG1* | Hearing loss | | [139] |
| Thr87Ile | *ACTG1* | Deafness, dominant progressive | | [140] |
| Val01Leu | *ACTB* | Baraitser-Winter syndrome | | [126] |
| Glu115Asp | *ACTB* | Neurodevelopmental disorder | | [141] |
| Glu115Lys | *ACTB* | Baraitser-Winter syndrome | SD1 | [142] |
| Lys116Asn | *ACTG1* | Deafness, dominant progressive | | [143] |
| Lys116Met | *ACTG1* | Deafness, dominant progressive | | [140] |
| Met117Thr | *ACTB* | Baraitser-Winter syndrome | | [126] |
| Thr118Ile | *ACTB* | Baraitser-Winter syndrome | | [137] |
| Thr118Ile | *ACTG1* | Baraitser-Winter syndrome | | [125] |
| Ile120Val | *ACTG1* | Deafness, dominant progressive | | [144] |
| Ala133Val | *ACTG1* | Baraitser-Winter syndrome | | [125] |
| Ser143Cys | *ACTG1* | Sensorineural deafness, nonsyndromic | | [145] |
| Thr147Ile | *ACTB* | Baraitser-Winter syndrome | | [126] |
| Met151Ile | *ACTG1* | Microlissencephaly | | [138] |
| Ser153Phe | *ACTG1* | Baraitser-Winter syndrome | | [125] |
| Thr161Ala | *ACTB* | Baraitser-Winter syndrome | | [146] |
| Thr161Met | *ACTG1* | Deafness | SD3 | [147] |
| Glu166Lys | *ACTG1* | Diaphragmatic hernia, congenital | | [148] |
| Leu169Phe | *ACTB* | Developmental delay, facial dysmorphia, ventricular arrhythmia and thrombocytopaenia | | [134] |
| Asp176Glu | *ACTB* | Epilepsy | | [149] |
| Asp176Tyr | *ACTG1* | Baraitser-Winter syndrome | | [124] |
| Ala179Gly | *ACTG1* | Hearing loss | | [139] |
| Arg181Tyr | *ACTB* | Developmental malformations, sensory hearing loss & dystonia | SD4 | [150] |
| Arg181Gln | *ACTG1* | Sensorineural deafness | | [145] |
| Asp185His | *ACTG1* | Hearing loss | | [151] |

| | | | | |
|---|---|---|---|---|
| Ile190Phe | *ACTG1* | Multiple congenital anomalies | | [152] |
| Arg194Cys | *ACTB* | Baraitser-Winter syndrome | | [125] |
| Arg194His | *ACTB* | Baraitser-Winter syndrome | | [125] |
| Arg194Ser | *ACTB* | Baraitser-Winter syndrome | | [126] |
| Thr201Lys | *ACTG1* | Baraitser-Winter syndrome | | [125] |
| Thr201Met | *ACTG1* | Agenesis of corpus callosum and neuronal heterotopia | | [153] |
| Ala202Gly | *ACTB* | Baraitser-Winter syndrome | | [126] |
| Arg202Gln | *ACTB* | Cerebral abnormalities | | [154] |
| Val207Leu | *ACTB* | Leukaemia, acute lymphoblastic | | [155] |
| Val207Met | *ACTB* | Baraitser-Winter syndrome | | [126] |
| Arg208Cys | *ACTG1* | Baraitser-Winter syndrome | | [156] |
| Lys211Arg | *ACTG1* | Hearing impairment, non-syndromic, autosomal dominant | | [157] |
| Glu239Lys | *ACTG1* | Deafness, dominant progressive | | [143] |
| Pro241Leu | *ACTG1* | Microlissencephaly | | [138] |
| Gly243Ser | *ACTB* | Baraitser-Winter syndrome | | [158] |
| Glu251Lys | *ACTG1* | Congenital heart disease | | [159] |
| Arg252Trp | *ACTG1* | Baraitser-Winter syndrome | | [125] |
| Arg254Trp | *ACTG1* | Baraitser-Winter syndrome | | [125] |
| Pro256Leu | *ACTG1* | Hearing impairment | | [160] |
| Pro262Leu | *ACTG1* | Deafness, dominant progressive | | [140] |
| Gly266Arg | *ACTB* | Baraitser-Winter syndrome | | [161] |
| Gly266Ser | *ACTG1* | Hearing loss, early-childhood | | [162] |
| Thr276Ile | *ACTG1* | Deafness, dominant progressive | SD3 | [163] |
| Met281Thr | *ACTG1* | Hearing loss, sensorineural | | [145] |
| Met281Val | *ACTG1* | Hearing loss | | [133] |
| Leu297Val | *ACTG1* | Deafness | | [164] |
| Met303Thr | *ACTG1* | Hearing loss, non-syndromic | | [165] |
| Met311Arg | *ACTB* | Developmental disability, microcephaly and thrombocytopenia | | [166] |
| Glu314Lys | *ACTG1* | Hearing loss | | [167] |
| Met323Lys | *ACTG1* | Hearing loss | | [168] |
| Ala329Val | *ACTB* | Developmental disability, microcephaly and thrombocytopenia | | [166] |
| Pro330Ala | *ACTG1* | Deafness, dominant progressive | | [140] |
| Pro330Ser | *ACTG1* | Hearing loss, sensorineural | | [169] |
| Glu332Gln | *ACTG1* | Baraitser-Winter syndrome | | [124] |
| Arg333His | *ACTG1* | Baraitser-Winter syndrome | | [124] |
| Leu348Met | *ACTG1* | Hearing loss | SD1 | [139] |
| Glu362Lys | *ACTB* | Neutrophil dysfunction and recurrent infection | | [170] |
| Ser363Leu | *ACTB* | Developmental disability, microcephaly and thrombocytopenia | | [166] |
| Val368Ala | *ACTG1* | Deafness, dominant progressive | | [171] |
| Lys370Term | *ACTB* | Developmental delay, intellectual disability and organ malformation | | [172] |

1c: Mutations in smooth ɣ actin

| Amino Acid Mutation* | Gene | Phenotype | SD | Ref. |
|---|---|---|---|---|
| Arg36His | *ACTG2* | Chronic intestinal pseudo obstruction | SD1 | [173] |
| Arg38Cys | *ACTG2* | MMIHS | | [174] |
| Arg38His | *ACTG2* | MMIHS | | [174] |
| Met43Thr | *ACTG2* | MMIHS | SD2 | [174] |
| Arg61Gln | *ACTG2* | MMIHS | | [175] |
| Arg61Gly | *ACTG2* | MMIHS | | [174] |
| Lys117Arg | *ACTG2* | Visceral myopathy, familial | SD1 | [174, 176] |
| Tyr132Asn | *ACTG2* | MMIHS | | [174] |
| Gly145Cys | *ACTG2* | Chronic intestinal pseudo obstruction | | [177] |
| Arg146Leu | *ACTG2* | Chronic intestinal pseudo obstruction | | [178] |
| Arg146Ser | *ACTG2* | Visceral myopathy, familial | | [179] |
| Arg176Cys | *ACTG2* | MMIHS | SD3 | [180] |
| Arg176His | *ACTG2* | MMIHS | | [174] |
| Arg176Leu | *ACTG2* | MMIHS | | [180] |
| Arg176Ser | *ACTG2* | MMIHS | | [181] |
| Thr193Ile | *ACTG2* | Chronic intestinal pseudo obstruction | SD4 | [182] |
| Gly196Asp | *ACTG2* | MMIHS | | [174] |
| Ala203Thr | *ACTG2* | Micro-colon megacystic syndrome | | [183] |
| Arg209Gln | *ACTG2* | Visceral myopathy, familial | | [184] |
| Arg209Term | *ACTG2* | Severe intestinal pseudo-obstruction | | [185] |
| Arg255Cys | *ACTG2* | MMIHS | | [174] |
| Arg255His | *ACTG2* | Visceral myopathy, familial | | [186] |

**Supplemental Table 1: Missense mutations in the Actin Genes.** The table provides the resulting amino acid substitution resulting from the mutation in the gene, the resulting disease, and the reference for each reported disease mutation. HCM: hypertrophic cardiomyopathy. DCM: Dilated Cardiomyopathy. MMIHS: Megacystis microcolon-intestinal hypoperistalsis syndrome. Database used: Human Genome Mutation Database. Accessed November 2019. Highlighted rows indicated by the red stars in Figure 1. *These aa numbers are the same as those in Figure 1. The first two residues (MC) are acetylated and cleaved for ɣ-smooth, $\alpha$-smooth, cardiac and skeletal actin, and the first residue (M) is acetylated and cleaved for ɣ- and β-actin. The numbering in Figure 1 ignores these first 1-2 residues. To aid in comparison (given the small difference in numbering between isoforms), the table is split into 3 sections (1a for $\alpha$-actin isoforms, 1b for β and ɣ-actin, and 1c for smooth ɣ actin). Small differences in numbering were accounted for in the analysis performed in Fig.2.

**Supplemental Figure 1.**

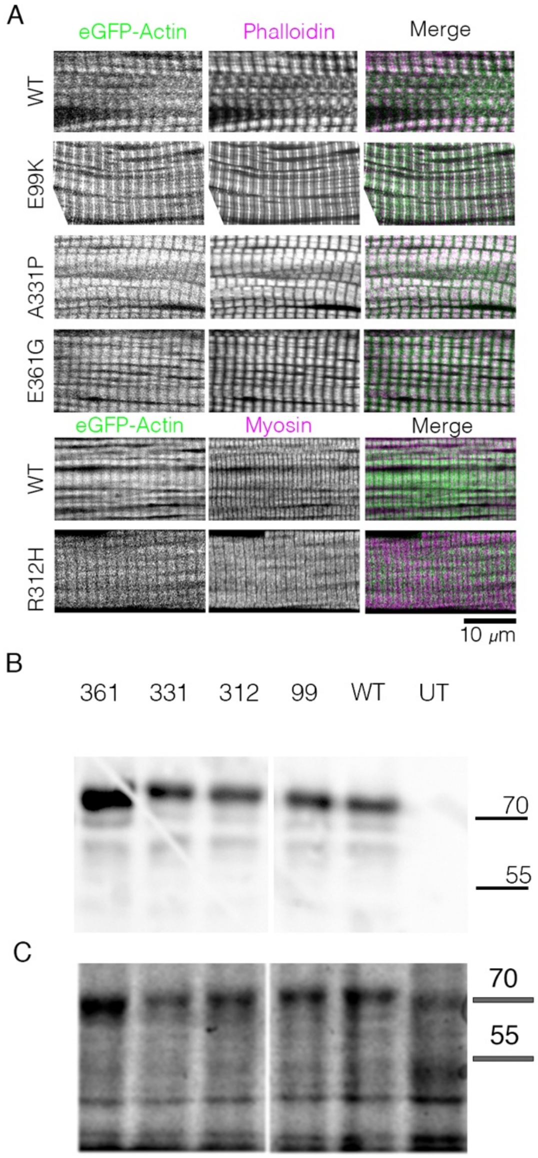


**Figure 3. Expression of wild type and mutant eGFP-tagged $\alpha$-cardiac actin.** A. shows the staining pattern for eGFP-cardiac actin (wild type and mutant isoforms) in adult rat cardiomyocytes, compared to the staining pattern for F-actin, using fluorescent phalloidin, or to that of myosin. To obtain these images, freshly isolated adult rat cardiomyocytes were cultured for 1-2 hours, and then treated with adenovirus expressing eGFP-actin constructs for up to 24 hours. Cells were fixed with fresh 4% paraformaldehyde in phosphate buffered saline, and then co-stained for actin, using fluorescent phalloidin, or for myosin, using A4.1025 (an antibody that recognises all striated myosin isoforms). Cells were imaged on a Zeiss LSM confocal using a 40x, 1.4N.A. oil objective. The image for E99K shows stronger actin staining at the Z-disc, compared to other samples. B. shows a western blot and C. the associated protein gel for equivalent samples to those shown in A. GFP-actin was visualised on the blot using an anti-eGFP antibody. Expression levels of the eGFP actin WT and mutant isoforms are approximately similar. Methods for these procedures are similar to previous work in our laboratory for eGFP-MHC, as described in [187].

## References


1. Kaindl, A. M.; Ruschendorf, F.; Krause, S.; Goebel, H. H.; Koehler, K.; Becker, C.; Pongratz, D.; Muller-Hocker, J.; Nurnberg, P.; Stoltenburg-Didinger, G.; Lochmuller, H.; Huebner, A., Missense mutations of ACTA1 cause dominant congenital myopathy with cores. *Journal of medical genetics* **2004,** 41, (11), 842-8.
2. Walsh, R.; Thomson, K. L.; Ware, J. S.; Funke, B. H.; Woodley, J.; McGuire, K. J.; Mazzarotto, F.; Blair, E.; Seller, A.; Taylor, J. C.; Minikel, E. V.; Exome Aggregation, C.; MacArthur, D. G.; Farrall, M.; Cook, S. A.; Watkins, H., Reassessment of Mendelian gene pathogenicity using 7,855 cardiomyopathy cases and 60,706 reference samples. *Genetics in medicine : official journal of the American College of Medical Genetics* **2017,** 19, (2), 192-203.
3. Overwater, E.; Marsili, L.; Baars, M. J. H.; Baas, A. F.; van de Beek, I.; Dulfer, E.; van Hagen, J. M.; Hilhorst-Hofstee, Y.; Kempers, M.; Krapels, I. P.; Menke, L. A.; Verhagen, J. M. A.; Yeung, K. K.; Zwijnenburg, P. J. G.; Groenink, M.; van Rijn, P.; Weiss, M. M.; Voorhoeve, E.; van Tintelen, J. P.; Houweling, A. C.; Maugeri, A., Results of next-generation sequencing gene panel diagnostics including copy-number variation analysis in 810 patients suspected of heritable thoracic aortic disorders. *Human mutation* **2018,** 39, (9), 1173-1192.
4. Laing, N. G.; Dye, D. E.; Wallgren-Pettersson, C.; Richard, G.; Monnier, N.; Lillis, S.; Winder, T. L.; Lochmuller, H.; Graziano, C.; Mitrani-Rosenbaum, S.; Twomey, D.; Sparrow, J. C.; Beggs, A. H.; Nowak, K. J., Mutations and polymorphisms of the skeletal muscle alpha-actin gene (ACTA1). *Human mutation* **2009,** 30, (9), 1267-77.
5. Kindel, S. J.; Miller, E. M.; Gupta, R.; Cripe, L. H.; Hinton, R. B.; Spicer, R. L.; Towbin, J. A.; Ware, S. M., Pediatric cardiomyopathy: importance of genetic and metabolic evaluation. *Journal of cardiac failure* **2012,** 18, (5), 396-403.
6. Nowak, K. J.; Wattanasirichaigoon, D.; Goebel, H. H.; Wilce, M.; Pelin, K.; Donner, K.; Jacob, R. L.; Hubner, C.; Oexle, K.; Anderson, J. R.; Verity, C. M.; North, K. N.; Iannaccone, S. T.; Muller, C. R.; Nurnberg, P.; Muntoni, F.; Sewry, C.; Hughes, I.; Sutphen, R.; Lacson, A. G.; Swoboda, K. J.; Vigneron, J.; Wallgren-Pettersson, C.; Beggs, A. H.; Laing, N. G., Mutations in the skeletal muscle alpha-actin gene in patients with actin myopathy and nemaline myopathy. *Nature genetics* **1999,** 23, (2), 208-12.
7. Ahmed, A. A.; Skaria, P.; Safina, N. P.; Thiffault, I.; Kats, A.; Taboada, E.; Habeebu, S.; Saunders, C., Arthrogryposis and pterygia as lethal end manifestations of genetically defined congenital myopathies. *American journal of medical genetics. Part A* **2018,** 176, (2), 359-367.
8. Stenzel, W.; Prokop, S.; Kress, W.; Huppmann, S.; Loui, A.; Sarioglu, N. M.; Laing, N. G.; Sparrow, J. C.; Heppner, F. L.; Goebel, H. H., Fetal akinesia caused by a novel actin filament aggregate myopathy skeletal muscle actin gene (ACTA1) mutation. *Neuromuscular disorders : NMD* **2010,** 20, (8), 531-3.
9. Regalado, E. S.; Guo, D. C.; Prakash, S.; Bensend, T. A.; Flynn, K.; Estrera, A.; Safi, H.; Liang, D.; Hyland, J.; Child, A.; Arno, G.; Boileau, C.; Jondeau, G.; Braverman, A.; Moran, R.; Morisaki, T.; Morisaki, H.; Pyeritz, R.; Coselli, J.; LeMaire, S.; Milewicz, D. M., Aortic Disease Presentation and Outcome Associated With ACTA2 Mutations. *Circulation. Cardiovascular genetics* **2015,** 8, (3), 457-64.
10. Frustaci, A.; De Luca, A.; Guida, V.; Biagini, T.; Mazza, T.; Gaudio, C.; Letizia, C.; Russo, M. A.; Galea, N.; Chimenti, C., Novel alpha-Actin Gene Mutation p.(Ala21Val) Causing Familial Hypertrophic Cardiomyopathy, Myocardial Noncompaction, and Transmural Crypts. Clinical-Pathologic Correlation. *Journal of the American Heart Association* **2018,** 7, (4).
11. Coppini, R.; Ho, C. Y.; Ashley, E.; Day, S.; Ferrantini, C.; Girolami, F.; Tomberli, B.; Bardi, S.; Torricelli, F.; Cecchi, F.; Mugelli, A.; Poggesi, C.; Tardiff, J.; Olivotto, I., Clinical phenotype

and outcome of hypertrophic cardiomyopathy associated with thin-filament gene mutations. *Journal of the American College of Cardiology* **2014,** 64, (24), 2589-2600.
12. Yoo, E. H.; Choi, S. H.; Jang, S. Y.; Suh, Y. L.; Lee, I.; Song, J. K.; Choe, Y. H.; Kim, J. W.; Ki, C. S.; Kim, D. K., Clinical, pathological, and genetic analysis of a Korean family with thoracic aortic aneurysms and dissections carrying a novel Asp26Tyr mutation. *Annals of clinical and laboratory science* **2010,** 40, (3), 278-84.
13. Sparrow, J. C.; Nowak, K. J.; Durling, H. J.; Beggs, A. H.; Wallgren-Pettersson, C.; Romero, N.; Nonaka, I.; Laing, N. G., Muscle disease caused by mutations in the skeletal muscle alpha-actin gene (ACTA1). *Neuromuscular disorders : NMD* **2003,** 13, (7-8), 519-31.
14. Poninska, J. K.; Bilinska, Z. T.; Franaszczyk, M.; Michalak, E.; Rydzanicz, M.; Szpakowski, E.; Pollak, A.; Milanowska, B.; Truszkowska, G.; Chmielewski, P.; Sioma, A.; Janaszek-Sitkowska, H.; Klisiewicz, A.; Michalowska, I.; Makowiecka-Ciesla, M.; Kolsut, P.; Stawinski, P.; Foss-Nieradko, B.; Szperl, M.; Grzybowski, J.; Hoffman, P.; Januszewicz, A.; Kusmierczyk, M.; Ploski, R., Next-generation sequencing for diagnosis of thoracic aortic aneurysms and dissections: diagnostic yield, novel mutations and genotype phenotype correlations. *Journal of translational medicine* **2016,** 14, (1), 115.
15. Renard, M.; Callewaert, B.; Baetens, M.; Campens, L.; MacDermot, K.; Fryns, J. P.; Bonduelle, M.; Dietz, H. C.; Gaspar, I. M.; Cavaco, D.; Stattin, E. L.; Schrander-Stumpel, C.; Coucke, P.; Loeys, B.; De Paepe, A.; De Backer, J., Novel MYH11 and ACTA2 mutations reveal a role for enhanced TGFbeta signaling in FTAAD. *International journal of cardiology* **2013,** 165, (2), 314-21.
16. Hoffjan, S.; Waldmuller, S.; Blankenfeldt, W.; Kotting, J.; Gehle, P.; Binner, P.; Epplen, J. T.; Scheffold, T., Three novel mutations in the ACTA2 gene in German patients with thoracic aortic aneurysms and dissections. *European journal of human genetics : EJHG* **2011,** 19, (5), 520-4.
17. Normand, E. A.; Braxton, A.; Nassef, S.; Ward, P. A.; Vetrini, F.; He, W.; Patel, V.; Qu, C.; Westerfield, L. E.; Stover, S.; Dharmadhikari, A. V.; Muzny, D. M.; Gibbs, R. A.; Dai, H.; Meng, L.; Wang, X.; Xiao, R.; Liu, P.; Bi, W.; Xia, F.; Walkiewicz, M.; Van den Veyver, I. B.; Eng, C. M.; Yang, Y., Clinical exome sequencing for fetuses with ultrasound abnormalities and a suspected Mendelian disorder. *Genome medicine* **2018,** 10, (1), 74.
18. Guo, D. C.; Papke, C. L.; Tran-Fadulu, V.; Regalado, E. S.; Avidan, N.; Johnson, R. J.; Kim, D. H.; Pannu, H.; Willing, M. C.; Sparks, E.; Pyeritz, R. E.; Singh, M. N.; Dalman, R. L.; Grotta, J. C.; Marian, A. J.; Boerwinkle, E. A.; Frazier, L. Q.; LeMaire, S. A.; Coselli, J. S.; Estrera, A. L.; Safi, H. J.; Veeraraghavan, S.; Muzny, D. M.; Wheeler, D. A.; Willerson, J. T.; Yu, R. K.; Shete, S. S.; Scherer, S. E.; Raman, C. S.; Buja, L. M.; Milewicz, D. M., Mutations in smooth muscle alpha-actin (ACTA2) cause coronary artery disease, stroke, and Moyamoya disease, along with thoracic aortic disease. *American journal of human genetics* **2009,** 84, (5), 617-27.
19. Zheng, J.; Guo, J.; Huang, L.; Wu, Q.; Yin, K.; Wang, L.; Zhang, T.; Quan, L.; Zhao, Q.; Cheng, J., Genetic diagnosis of acute aortic dissection in South China Han population using next-generation sequencing. *International journal of legal medicine* **2018,** 132, (5), 1273-1280.
20. Proost, D.; Vandeweyer, G.; Meester, J. A.; Salemink, S.; Kempers, M.; Ingram, C.; Peeters, N.; Saenen, J.; Vrints, C.; Lacro, R. V.; Roden, D.; Wuyts, W.; Dietz, H. C.; Mortier, G.; Loeys, B. L.; Van Laer, L., Performant Mutation Identification Using Targeted Next-Generation Sequencing of 14 Thoracic Aortic Aneurysm Genes. *Human mutation* **2015,** 36, (8), 808-14.
21. Wallgren-Pettersson, C.; Laing, N. G., Report of the 83rd ENMC International Workshop: 4th Workshop on Nemaline Myopathy, 22-24 September 2000, Naarden, The Netherlands. *Neuromuscular disorders : NMD* **2001,** 11, (6-7), 589-95.
22. Zou, Y.; Wang, J.; Liu, X.; Wang, Y.; Chen, Y.; Sun, K.; Gao, S.; Zhang, C.; Wang, Z.; Zhang, Y.; Feng, X.; Song, Y.; Wu, Y.; Zhang, H.; Jia, L.; Wang, H.; Wang, D.; Yan, C.; Lu, M.; Zhou, X.; Song, L.; Hui, R., Multiple gene mutations, not the type of mutation, are the modifier of left ventricle hypertrophy in patients with hypertrophic cardiomyopathy. *Molecular biology reports* **2013,** 40, (6), 3969-76.

23. Disabella, E.; Grasso, M.; Gambarin, F. I.; Narula, N.; Dore, R.; Favalli, V.; Serio, A.; Antoniazzi, E.; Mosconi, M.; Pasotti, M.; Odero, A.; Arbustini, E., Risk of dissection in thoracic aneurysms associated with mutations of smooth muscle alpha-actin 2 (ACTA2). *Heart (British Cardiac Society)* **2011,** 97, (4), 321-6.
24. Zhang, A.; Jo, A.; Grajewski, K.; Kim, J., Characteristic Cerebrovascular Findings Associated with ACTA2 Gene Mutations. *The Canadian journal of neurological sciences. Le journal canadien des sciences neurologiques* **2019,** 46, (3), 342-343.
25. Tadokoro, K.; Ohta, Y.; Sasaki, R.; Takahashi, Y.; Sato, K.; Shang, J.; Takemoto, M.; Hishikawa, N.; Yamashita, T.; Nakamura, K.; Nishino, I.; Abe, K., Congenital myopathy with fiber-type disproportion accompanied by dilated cardiomyopathy in a patient with a novel p.G48A ACTA1 mutation. *Journal of the neurological sciences* **2018,** 393, 142-144.
26. Witting, N.; Werlauff, U.; Duno, M.; Vissing, J., Prevalence and phenotypes of congenital myopathy due to alpha-actin 1 gene mutations. *Muscle & nerve* **2016,** 53, (3), 388-93.
27. Liewluck, T.; Niu, Z.; Moore, S. A.; Alsharabati, M.; Milone, M., ACTA1-myopathy with prominent finger flexor weakness and rimmed vacuoles. *Neuromuscular disorders : NMD* **2019,** 29, (5), 388-391.
28. Tominaga, K.; Hayashi, Y. K.; Goto, K.; Minami, N.; Noguchi, S.; Nonaka, I.; Miki, T.; Nishino, I., Congenital myotonic dystrophy can show congenital fiber type disproportion pathology. *Acta neuropathologica* **2010,** 119, (4), 481-6.
29. Hayashi, T.; Tanimoto, K.; Hirayama-Yamada, K.; Tsuda, E.; Ayusawa, M.; Nunoda, S.; Hosaki, A.; Kimura, A., Genetic background of Japanese patients with pediatric hypertrophic and restrictive cardiomyopathy. *Journal of human genetics* **2018,** 63, (9), 989-996.
30. Wang, J.; Wang, Y.; Zou, Y.; Sun, K.; Wang, Z.; Ding, H.; Yuan, J.; Wei, W.; Hou, Q.; Wang, H.; Liu, X.; Zhang, H.; Ji, Y.; Zhou, X.; Sharma, R. K.; Wang, D.; Ahmad, F.; Hui, R.; Song, L., Malignant effects of multiple rare variants in sarcomere genes on the prognosis of patients with hypertrophic cardiomyopathy. *European journal of heart failure* **2014,** 16, (9), 950-7.
31. Ilkovski, B.; Nowak, K. J.; Domazetovska, A.; Maxwell, A. L.; Clement, S.; Davies, K. E.; Laing, N. G.; North, K. N.; Cooper, S. T., Evidence for a dominant-negative effect in ACTA1 nemaline myopathy caused by abnormal folding, aggregation and altered polymerization of mutant actin isoforms. *Human molecular genetics* **2004,** 13, (16), 1727-43.
32. Chae, J. H.; Vasta, V.; Cho, A.; Lim, B. C.; Zhang, Q.; Eun, S. H.; Hahn, S. H., Utility of next generation sequencing in genetic diagnosis of early onset neuromuscular disorders. *Journal of medical genetics* **2015,** 52, (3), 208-16.
33. Graziano, C.; Bertini, E.; Porfirio, B., De novo alpha-actin mutations in monozygotic twins. *Clinical genetics* **2005,** 68, (1), 91-2.
34. Prevalence and architecture of de novo mutations in developmental disorders. *Nature* **2017,** 542, (7642), 433-438.
35. Augiere, C.; Megy, S.; El Malti, R.; Boland, A.; El Zein, L.; Verrier, B.; Megarbane, A.; Deleuze, J. F.; Bouvagnet, P., A Novel Alpha Cardiac Actin (ACTC1) Mutation Mapping to a Domain in Close Contact with Myosin Heavy Chain Leads to a Variety of Congenital Heart Defects, Arrhythmia and Possibly Midline Defects. *PloS one* **2015,** 10, (6), e0127903.
36. Agrawal, P. B.; Strickland, C. D.; Midgett, C.; Morales, A.; Newburger, D. E.; Poulos, M. A.; Tomczak, K. K.; Ryan, M. M.; Iannaccone, S. T.; Crawford, T. O.; Laing, N. G.; Beggs, A. H., Heterogeneity of nemaline myopathy cases with skeletal muscle alpha-actin gene mutations. *Annals of neurology* **2004,** 56, (1), 86-96.
37. Morita, H.; Rehm, H. L.; Menesses, A.; McDonough, B.; Roberts, A. E.; Kucherlapati, R.; Towbin, J. A.; Seidman, J. G.; Seidman, C. E., Shared genetic causes of cardiac hypertrophy in children and adults. *The New England journal of medicine* **2008,** 358, (18), 1899-908.
38. Burns, C.; Bagnall, R. D.; Lam, L.; Semsarian, C.; Ingles, J., Multiple Gene Variants in Hypertrophic Cardiomyopathy in the Era of Next-Generation Sequencing. *Circulation. Cardiovascular genetics* **2017,** 10, (4).

39. Wang, C.; Hata, Y.; Hirono, K.; Takasaki, A.; Ozawa, S. W.; Nakaoka, H.; Saito, K.; Miyao, N.; Okabe, M.; Ibuki, K.; Nishida, N.; Origasa, H.; Yu, X.; Bowles, N. E.; Ichida, F., A Wide and Specific Spectrum of Genetic Variants and Genotype-Phenotype Correlations Revealed by Next-Generation Sequencing in Patients with Left Ventricular Noncompaction. *Journal of the American Heart Association* **2017,** 6, (9).
40. Saito, Y.; Komaki, H.; Hattori, A.; Takeuchi, F.; Sasaki, M.; Kawabata, K.; Mitsuhashi, S.; Tominaga, K.; Hayashi, Y. K.; Nowak, K. J.; Laing, N. G.; Nonaka, I.; Nishino, I., Extramuscular manifestations in children with severe congenital myopathy due to ACTA1 gene mutations. *Neuromuscular disorders : NMD* **2011,** 21, (7), 489-93.
41. Richard, P.; Ader, F.; Roux, M.; Donal, E.; Eicher, J. C.; Aoutil, N.; Huttin, O.; Selton-Suty, C.; Coisne, D.; Jondeau, G.; Damy, T.; Mansencal, N.; Casalta, A. C.; Michel, N.; Haentjens, J.; Faivre, L.; Lavoute, C.; Nguyen, K.; Tregouet, D. A.; Habib, G.; Charron, P., Targeted panel sequencing in adult patients with left ventricular non-compaction reveals a large genetic heterogeneity. *Clinical genetics* **2019,** 95, (3), 356-367.
42. Olson, T. M.; Doan, T. P.; Kishimoto, N. Y.; Whitby, F. G.; Ackerman, M. J.; Fananapazir, L., Inherited and de novo mutations in the cardiac actin gene cause hypertrophic cardiomyopathy. *Journal of molecular and cellular cardiology* **2000,** 32, (9), 1687-94.
43. Bee, K. J.; Wilkes, D. C.; Devereux, R. B.; Basson, C. T.; Hatcher, C. J., TGFbetaRIIb mutations trigger aortic aneurysm pathogenesis by altering transforming growth factor beta2 signal transduction. *Circulation. Cardiovascular genetics* **2012,** 5, (6), 621-9.
44. Miszalski-Jamka, K.; Jefferies, J. L.; Mazur, W.; Glowacki, J.; Hu, J.; Lazar, M.; Gibbs, R. A.; Liczko, J.; Klys, J.; Venner, E.; Muzny, D. M.; Rycaj, J.; Bialkowski, J.; Kluczewska, E.; Kalarus, Z.; Jhangiani, S.; Al-Khalidi, H.; Kukulski, T.; Lupski, J. R.; Craigen, W. J.; Bainbridge, M. N., Novel Genetic Triggers and Genotype-Phenotype Correlations in Patients With Left Ventricular Noncompaction. *Circulation. Cardiovascular genetics* **2017,** 10, (4).
45. Ladha, S.; Coons, S.; Johnsen, S.; Sambuughin, N.; Bien-Wilner, R.; Sivakumar, K., Histopathologic progression and a novel mutation in a child with nemaline myopathy. *Journal of child neurology* **2008,** 23, (7), 813-7.
46. Lerner-Ellis, J. P.; Aldubayan, S. H.; Hernandez, A. L.; Kelly, M. A.; Stuenkel, A. J.; Walsh, J.; Joshi, V. A., The spectrum of FBN1, TGFbetaR1, TGFbetaR2 and ACTA2 variants in 594 individuals with suspected Marfan Syndrome, Loeys-Dietz Syndrome or Thoracic Aortic Aneurysms and Dissections (TAAD). *Molecular genetics and metabolism* **2014,** 112, (2), 171-6.
47. Punetha, J.; Kesari, A.; Uapinyoying, P.; Giri, M.; Clarke, N. F.; Waddell, L. B.; North, K. N.; Ghaoui, R.; O'Grady, G. L.; Oates, E. C.; Sandaradura, S. A.; Bonnemann, C. G.; Donkervoort, S.; Plotz, P. H.; Smith, E. C.; Tesi-Rocha, C.; Bertorini, T. E.; Tarnopolsky, M. A.; Reitter, B.; Hausmanowa-Petrusewicz, I.; Hoffman, E. P., Targeted Re-Sequencing Emulsion PCR Panel for Myopathies: Results in 94 Cases. *Journal of neuromuscular diseases* **2016,** 3, (2), 209-225.
48. Ke, T.; Han, M.; Zhao, M.; Wang, Q. K.; Zhang, H.; Zhao, Y.; Ruan, X.; Li, H.; Xu, C.; Sun, T., Alpha-actin-2 mutations in Chinese patients with a non-syndromatic thoracic aortic aneurysm. *BMC medical genetics* **2016,** 17, (1), 45.
49. Guo, D. C.; Pannu, H.; Tran-Fadulu, V.; Papke, C. L.; Yu, R. K.; Avidan, N.; Bourgeois, S.; Estrera, A. L.; Safi, H. J.; Sparks, E.; Amor, D.; Ades, L.; McConnell, V.; Willoughby, C. E.; Abuelo, D.; Willing, M.; Lewis, R. A.; Kim, D. H.; Scherer, S.; Tung, P. P.; Ahn, C.; Buja, L. M.; Raman, C. S.; Shete, S. S.; Milewicz, D. M., Mutations in smooth muscle alpha-actin (ACTA2) lead to thoracic aortic aneurysms and dissections. *Nature genetics* **2007,** 39, (12), 1488-93.
50. Joureau, B.; de Winter, J. M.; Conijn, S.; Bogaards, S. J. P.; Kovacevic, I.; Kalganov, A.; Persson, M.; Lindqvist, J.; Stienen, G. J. M.; Irving, T. C.; Ma, W.; Yuen, M.; Clarke, N. F.; Rassier, D. E.; Malfatti, E.; Romero, N. B.; Beggs, A. H.; Ottenheijm, C. A. C., Dysfunctional sarcomere contractility contributes to muscle weakness in ACTA1-related nemaline myopathy (NEM3). *Annals of neurology* **2018,** 83, (2), 269-282.

51. Zimmerman, R. S.; Cox, S.; Lakdawala, N. K.; Cirino, A.; Mancini-DiNardo, D.; Clark, E.; Leon, A.; Duffy, E.; White, E.; Baxter, S.; Alaamery, M.; Farwell, L.; Weiss, S.; Seidman, C. E.; Seidman, J. G.; Ho, C. Y.; Rehm, H. L.; Funke, B. H., A novel custom resequencing array for dilated cardiomyopathy. *Genetics in medicine : official journal of the American College of Medical Genetics* **2010,** 12, (5), 268-78.
52. Lopes, L. R.; Syrris, P.; Guttmann, O. P.; O'Mahony, C.; Tang, H. C.; Dalageorgou, C.; Jenkins, S.; Hubank, M.; Monserrat, L.; McKenna, W. J.; Plagnol, V.; Elliott, P. M., Novel genotype-phenotype associations demonstrated by high-throughput sequencing in patients with hypertrophic cardiomyopathy. *Heart (British Cardiac Society)* **2015,** 101, (4), 294-301.
53. Matsson, H.; Eason, J.; Bookwalter, C. S.; Klar, J.; Gustavsson, P.; Sunnegardh, J.; Enell, H.; Jonzon, A.; Vikkula, M.; Gutierrez, I.; Granados-Riveron, J.; Pope, M.; Bu'Lock, F.; Cox, J.; Robinson, T. E.; Song, F.; Brook, D. J.; Marston, S.; Trybus, K. M.; Dahl, N., Alpha-cardiac actin mutations produce atrial septal defects. *Human molecular genetics* **2008,** 17, (2), 256-65.
54. Lakdawala, N. K.; Funke, B. H.; Baxter, S.; Cirino, A. L.; Roberts, A. E.; Judge, D. P.; Johnson, N.; Mendelsohn, N. J.; Morel, C.; Care, M.; Chung, W. K.; Jones, C.; Psychogios, A.; Duffy, E.; Rehm, H. L.; White, E.; Seidman, J. G.; Seidman, C. E.; Ho, C. Y., Genetic testing for dilated cardiomyopathy in clinical practice. *Journal of cardiac failure* **2012,** 18, (4), 296-303.
55. Fang, M.; Yu, C.; Chen, S.; Xiong, W.; Li, X.; Zeng, R.; Zhuang, J.; Fan, R., Identification of Novel Clinically Relevant Variants in 70 Southern Chinese patients with Thoracic Aortic Aneurysm and Dissection by Next-generation Sequencing. *Scientific reports* **2017,** 7, (1), 10035.
56. Ilkovski, B.; Cooper, S. T.; Nowak, K.; Ryan, M. M.; Yang, N.; Schnell, C.; Durling, H. J.; Roddick, L. G.; Wilkinson, I.; Kornberg, A. J.; Collins, K. J.; Wallace, G.; Gunning, P.; Hardeman, E. C.; Laing, N. G.; North, K. N., Nemaline myopathy caused by mutations in the muscle alpha-skeletal-actin gene. *American journal of human genetics* **2001,** 68, (6), 1333-43.
57. Koy, A.; Ilkovski, B.; Laing, N.; North, K.; Weis, J.; Neuen-Jacob, E.; Mayatepek, E.; Voit, T., Nemaline myopathy with exclusively intranuclear rods and a novel mutation in ACTA1 (Q139H). *Neuropediatrics* **2007,** 38, (6), 282-6.
58. Arai, A.; Mitsuhashi, S.; Saito, Y.; Komaki, H.; Sakuma, H.; Nakagawa, E.; Sugai, K.; Sasaki, M.; Robertson, S. P.; Nishimura, G.; Yamamoto, T.; Nonaka, I.; Nishino, I., Nemaline (actin) myopathy with myofibrillar dysgenesis and abnormal ossification. *Neuromuscular disorders : NMD* **2009,** 19, (7), 485-8.
59. Morisaki, H.; Akutsu, K.; Ogino, H.; Kondo, N.; Yamanaka, I.; Tsutsumi, Y.; Yoshimuta, T.; Okajima, T.; Matsuda, H.; Minatoya, K.; Sasaki, H.; Tanaka, H.; Ishibashi-Ueda, H.; Morisaki, T., Mutation of ACTA2 gene as an important cause of familial and nonfamilial nonsyndromatic thoracic aortic aneurysm and/or dissection (TAAD). *Human mutation* **2009,** 30, (10), 1406-11.
60. Ghaoui, R.; Cooper, S. T.; Lek, M.; Jones, K.; Corbett, A.; Reddel, S. W.; Needham, M.; Liang, C.; Waddell, L. B.; Nicholson, G.; O'Grady, G.; Kaur, S.; Ong, R.; Davis, M.; Sue, C. M.; Laing, N. G.; North, K. N.; MacArthur, D. G.; Clarke, N. F., Use of Whole-Exome Sequencing for Diagnosis of Limb-Girdle Muscular Dystrophy: Outcomes and Lessons Learned. *JAMA neurology* **2015,** 72, (12), 1424-32.
61. Miyatake, S.; Koshimizu, E.; Hayashi, Y. K.; Miya, K.; Shiina, M.; Nakashima, M.; Tsurusaki, Y.; Miyake, N.; Saitsu, H.; Ogata, K.; Nishino, I.; Matsumoto, N., Deep sequencing detects very-low-grade somatic mosaicism in the unaffected mother of siblings with nemaline myopathy. *Neuromuscular disorders : NMD* **2014,** 24, (7), 642-7.
62. Kawase, K.; Nishino, I.; Sugimoto, M.; Kouwaki, M.; Koyama, N.; Yokochi, K., Hypoxic ischemic encephalopathy in a case of intranuclear rod myopathy without any prenatal sentinel event. *Brain & development* **2015,** 37, (2), 265-9.
63. Ravenscroft, G.; Wilmshurst, J. M.; Pillay, K.; Sivadorai, P.; Wallefeld, W.; Nowak, K. J.; Laing, N. G., A novel ACTA1 mutation resulting in a severe congenital myopathy with

nemaline bodies, intranuclear rods and type I fibre predominance. *Neuromuscular disorders : NMD* **2011,** 21, (1), 31-6.
64. O'Grady, G. L.; Best, H. A.; Oates, E. C.; Kaur, S.; Charlton, A.; Brammah, S.; Punetha, J.; Kesari, A.; North, K. N.; Ilkovski, B.; Hoffman, E. P.; Clarke, N. F., Recessive ACTA1 variant causes congenital muscular dystrophy with rigid spine. *European journal of human genetics : EJHG* **2015,** 23, (6), 883-6.
65. Sakai, H.; Suzuki, S.; Mizuguchi, T.; Imoto, K.; Yamashita, Y.; Doi, H.; Kikuchi, M.; Tsurusaki, Y.; Saitsu, H.; Miyake, N.; Masuda, M.; Matsumoto, N., Rapid detection of gene mutations responsible for non-syndromic aortic aneurysm and dissection using two different methods: resequencing microarray technology and next-generation sequencing. *Human genetics* **2012,** 131, (4), 591-9.
66. Mogensen, J.; Perrot, A.; Andersen, P. S.; Havndrup, O.; Klausen, I. C.; Christiansen, M.; Bross, P.; Egeblad, H.; Bundgaard, H.; Osterziel, K. J.; Haltern, G.; Lapp, H.; Reinecke, P.; Gregersen, N.; Borglum, A. D., Clinical and genetic characteristics of alpha cardiac actin gene mutations in hypertrophic cardiomyopathy. *Journal of medical genetics* **2004,** 41, (1), e10.
67. Vasilescu, C.; Ojala, T. H.; Brilhante, V.; Ojanen, S.; Hinterding, H. M.; Palin, E.; Alastalo, T. P.; Koskenvuo, J.; Hiippala, A.; Jokinen, E.; Jahnukainen, T.; Lohi, J.; Pihkala, J.; Tyni, T. A.; Carroll, C. J.; Suomalainen, A., Genetic Basis of Severe Childhood-Onset Cardiomyopathies. *Journal of the American College of Cardiology* **2018,** 72, (19), 2324-2338.
68. Greenway, S. C.; McLeod, R.; Hume, S.; Roslin, N. M.; Alvarez, N.; Giuffre, M.; Zhan, S. H.; Shen, Y.; Preuss, C.; Andelfinger, G.; Jones, S. J.; Gerull, B., Exome sequencing identifies a novel variant in ACTC1 associated with familial atrial septal defect. *The Canadian journal of cardiology* **2014,** 30, (2), 181-7.
69. Meuwissen, M. E.; Lequin, M. H.; Bindels-de Heus, K.; Bruggenwirth, H. T.; Knapen, M. F.; Dalinghaus, M.; de Coo, R.; van Bever, Y.; Winkelman, B. H.; Mancini, G. M., ACTA2 mutation with childhood cardiovascular, autonomic and brain anomalies and severe outcome. *American journal of medical genetics. Part A* **2013,** 161a, (6), 1376-80.
70. Milewicz, D. M.; Ostergaard, J. R.; Ala-Kokko, L. M.; Khan, N.; Grange, D. K.; Mendoza-Londono, R.; Bradley, T. J.; Olney, A. H.; Ades, L.; Maher, J. F.; Guo, D.; Buja, L. M.; Kim, D.; Hyland, J. C.; Regalado, E. S., De novo ACTA2 mutation causes a novel syndrome of multisystemic smooth muscle dysfunction. *American journal of medical genetics. Part A* **2010,** 152a, (10), 2437-43.
71. Munot, P.; Saunders, D. E.; Milewicz, D. M.; Regalado, E. S.; Ostergaard, J. R.; Braun, K. P.; Kerr, T.; Lichtenbelt, K. D.; Philip, S.; Rittey, C.; Jacques, T. S.; Cox, T. C.; Ganesan, V., A novel distinctive cerebrovascular phenotype is associated with heterozygous Arg179 ACTA2 mutations. *Brain : a journal of neurology* **2012,** 135, (Pt 8), 2506-14.
72. Winter, J. M.; Joureau, B.; Lee, E. J.; Kiss, B.; Yuen, M.; Gupta, V. A.; Pappas, C. T.; Gregorio, C. C.; Stienen, G. J.; Edvardson, S.; Wallgren-Pettersson, C.; Lehtokari, V. L.; Pelin, K.; Malfatti, E.; Romero, N. B.; Engelen, B. G.; Voermans, N. C.; Donkervoort, S.; Bonnemann, C. G.; Clarke, N. F.; Beggs, A. H.; Granzier, H.; Ottenheijm, C. A., Mutation-specific effects on thin filament length in thin filament myopathy. *Annals of neurology* **2016,** 79, (6), 959-69.
73. Wooderchak-Donahue, W.; VanSant-Webb, C.; Tvrdik, T.; Plant, P.; Lewis, T.; Stocks, J.; Raney, J. A.; Meyers, L.; Berg, A.; Rope, A. F.; Yetman, A. T.; Bleyl, S. B.; Mesley, R.; Bull, D. A.; Collins, R. T.; Ojeda, M. M.; Roberts, A.; Lacro, R.; Woerner, A.; Stoler, J.; Bayrak-Toydemir, P., Clinical utility of a next generation sequencing panel assay for Marfan and Marfan-like syndromes featuring aortopathy. *American journal of medical genetics. Part A* **2015,** 167a, (8), 1747-57.
74. Stehlikova, K.; Skalova, D.; Zidkova, J.; Haberlova, J.; Vohanka, S.; Mazanec, R.; Mrazova, L.; Vondracek, P.; Oslejskova, H.; Zamecnik, J.; Honzik, T.; Zeman, J.; Magner, M.; Siskova, D.; Langova, M.; Gregor, V.; Godava, M.; Smolka, V.; Fajkusova, L., Muscular dystrophies and

myopathies: the spectrum of mutated genes in the Czech Republic. *Clinical genetics* **2017,** 91, (3), 463-469.
75. Hernandez-Lain, A.; Cantero, D.; Camacho-Salas, A.; Toldos, O.; Esteban, I.; Pascual, I.; Dominguez-Gonzalez, C., Autosomal dominant distal myopathy with nemaline rods due to p.Glu197Asp mutation in ACTA1. *Neuromuscular disorders : NMD* **2019,** 29, (3), 247-250.
76. Castiglioni, C.; Cassandrini, D.; Fattori, F.; Bellacchio, E.; D'Amico, A.; Alvarez, K.; Gejman, R.; Diaz, J.; Santorelli, F. M.; Romero, N. B.; Bertini, E.; Bevilacqua, J. A., Muscle magnetic resonance imaging and histopathology in ACTA1-related congenital nemaline myopathy. *Muscle & nerve* **2014,** 50, (6), 1011-6.
77. Feng, J. J.; Marston, S., Genotype-phenotype correlations in ACTA1 mutations that cause congenital myopathies. *Neuromuscular disorders : NMD* **2009,** 19, (1), 6-16.
78. Dal Ferro, M.; Stolfo, D.; Altinier, A.; Gigli, M.; Perrieri, M.; Ramani, F.; Barbati, G.; Pivetta, A.; Brun, F.; Monserrat, L.; Giacca, M.; Mestroni, L.; Merlo, M.; Sinagra, G., Association between mutation status and left ventricular reverse remodelling in dilated cardiomyopathy. *Heart (British Cardiac Society)* **2017,** 103, (21), 1704-1710.
79. Graziano, C.; Bertini, E.; Minetti, C.; Porfirio, B., Alpha-actin gene mutations and polymorphisms in Italian patients with nemaline myopathy. *International journal of molecular medicine* **2004,** 13, (6), 805-9.
80. Meng, L.; Pammi, M.; Saronwala, A.; Magoulas, P.; Ghazi, A. R.; Vetrini, F.; Zhang, J.; He, W.; Dharmadhikari, A. V.; Qu, C.; Ward, P.; Braxton, A.; Narayanan, S.; Ge, X.; Tokita, M. J.; Santiago-Sim, T.; Dai, H.; Chiang, T.; Smith, H.; Azamian, M. S.; Robak, L.; Bostwick, B. L.; Schaaf, C. P.; Potocki, L.; Scaglia, F.; Bacino, C. A.; Hanchard, N. A.; Wangler, M. F.; Scott, D.; Brown, C.; Hu, J.; Belmont, J. W.; Burrage, L. C.; Graham, B. H.; Sutton, V. R.; Craigen, W. J.; Plon, S. E.; Lupski, J. R.; Beaudet, A. L.; Gibbs, R. A.; Muzny, D. M.; Miller, M. J.; Wang, X.; Leduc, M. S.; Xiao, R.; Liu, P.; Shaw, C.; Walkiewicz, M.; Bi, W.; Xia, F.; Lee, B.; Eng, C. M.; Yang, Y.; Lalani, S. R., Use of Exome Sequencing for Infants in Intensive Care Units: Ascertainment of Severe Single-Gene Disorders and Effect on Medical Management. *JAMA pediatrics* **2017,** 171, (12), e173438.
81. Takasaki, A.; Hirono, K.; Hata, Y.; Wang, C.; Takeda, M.; Yamashita, J. K.; Chang, B.; Nakaoka, H.; Okabe, M.; Miyao, N.; Saito, K.; Ibuki, K.; Ozawa, S.; Sekine, M.; Yoshimura, N.; Nishida, N.; Bowles, N. E.; Ichida, F., Sarcomere gene variants act as a genetic trigger underlying the development of left ventricular noncompaction. *Pediatric research* **2018,** 84, (5), 733-742.
82. Vill, K.; Blaschek, A.; Glaser, D.; Kuhn, M.; Haack, T.; Alhaddad, B.; Wagner, M.; Kovacs-Nagy, R.; Tacke, M.; Gerstl, L.; Schroeder, A. S.; Borggraefe, I.; Mueller, C.; Schlotter-Weigel, B.; Schoser, B.; Walter, M. C.; Muller-Felber, W., Early-Onset Myopathies: Clinical Findings, Prevalence of Subgroups and Diagnostic Approach in a Single Neuromuscular Referral Center in Germany. *Journal of neuromuscular diseases* **2017,** 4, (4), 315-325.
83. Yoshida, Y.; Hirono, K.; Nakamura, K.; Suzuki, T.; Hata, Y.; Nishida, N., A novel ACTC1 mutation in a young boy with left ventricular noncompaction and arrhythmias. *HeartRhythm case reports* **2016,** 2, (1), 92-97.
84. Aljeaid, D.; Sanchez, A. I.; Wakefield, E.; Chadwell, S. E.; Moore, N.; Prada, C. E.; Zhang, W., Prevalence of pathogenic and likely pathogenic variants in the RASopathy genes in patients who have had panel testing for cardiomyopathy. *American journal of medical genetics. Part A* **2019,** 179, (4), 608-614.
85. Van Driest, S. L.; Ellsworth, E. G.; Ommen, S. R.; Tajik, A. J.; Gersh, B. J.; Ackerman, M. J., Prevalence and spectrum of thin filament mutations in an outpatient referral population with hypertrophic cardiomyopathy. *Circulation* **2003,** 108, (4), 445-51.
86. Cecconi, M.; Parodi, M. I.; Formisano, F.; Spirito, P.; Autore, C.; Musumeci, M. B.; Favale, S.; Forleo, C.; Rapezzi, C.; Biagini, E.; Davi, S.; Canepa, E.; Pennese, L.; Castagnetta, M.; Degiorgio, D.; Coviello, D. A., Targeted next-generation sequencing helps to decipher the

genetic and phenotypic heterogeneity of hypertrophic cardiomyopathy. *International journal of molecular medicine* **2016,** 38, (4), 1111-24.
87. Kim, S. Y.; Park, Y. E.; Kim, H. S.; Lee, C. H.; Yang, D. H.; Kim, D. S., Nemaline myopathy and non-fatal hypertrophic cardiomyopathy caused by a novel ACTA1 E239K mutation. *Journal of the neurological sciences* **2011,** 307, (1-2), 171-3.
88. Guglieri, M.; Sambuughin, N.; Sarkozy, A.; Barresi, R.; Lochmüller, H.; Bushby, K.; Goldfarb, L. G.; Straub, V., A.P.6 : Autosomal recessive myofibrillar myopathy caused by ACTA1 mutations. *Neuromuscular Disorders* **2014,** 24, (9), 832.
89. Liewluck, T.; Sorenson, E. J.; Walkiewicz, M. A.; Rumilla, K. M.; Milone, M., Autosomal dominant distal myopathy due to a novel ACTA1 mutation. *Neuromuscular disorders : NMD* **2017,** 27, (8), 742-746.
90. Reza, N.; Garg, A.; Merrill, S. L.; Chowns, J. L.; Rao, S.; Owens, A. T., ACTA1 Novel Likely Pathogenic Variant in a Family With Dilated Cardiomyopathy. *Circulation. Genomic and precision medicine* **2018,** 11, (10), e002243.
91. Weerakkody, R.; Ross, D.; Parry, D. A.; Ziganshin, B.; Vandrovcova, J.; Gampawar, P.; Abdullah, A.; Biggs, J.; Dumfarth, J.; Ibrahim, Y.; Bicknell, C.; Field, M.; Elefteriades, J.; Cheshire, N.; Aitman, T. J., Targeted genetic analysis in a large cohort of familial and sporadic cases of aneurysm or dissection of the thoracic aorta. *Genetics in medicine : official journal of the American College of Medical Genetics* **2018,** 20, (11), 1414-1422.
92. Maggi, L.; Scoto, M.; Cirak, S.; Robb, S. A.; Klein, A.; Lillis, S.; Cullup, T.; Feng, L.; Manzur, A. Y.; Sewry, C. A.; Abbs, S.; Jungbluth, H.; Muntoni, F., Congenital myopathies--clinical features and frequency of individual subtypes diagnosed over a 5-year period in the United Kingdom. *Neuromuscular disorders : NMD* **2013,** 23, (3), 195-205.
93. Pugh, T. J.; Kelly, M. A.; Gowrisankar, S.; Hynes, E.; Seidman, M. A.; Baxter, S. M.; Bowser, M.; Harrison, B.; Aaron, D.; Mahanta, L. M.; Lakdawala, N. K.; McDermott, G.; White, E. T.; Rehm, H. L.; Lebo, M.; Funke, B. H., The landscape of genetic variation in dilated cardiomyopathy as surveyed by clinical DNA sequencing. *Genetics in medicine : official journal of the American College of Medical Genetics* **2014,** 16, (8), 601-8.
94. Ohlsson, M.; Tajsharghi, H.; Darin, N.; Kyllerman, M.; Oldfors, A., Follow-up of nemaline myopathy in two patients with novel mutations in the skeletal muscle alpha-actin gene (ACTA1). *Neuromuscular disorders : NMD* **2004,** 14, (8-9), 471-5.
95. Jungbluth, H.; Sewry, C. A.; Brown, S. C.; Nowak, K. J.; Laing, N. G.; Wallgren-Pettersson, C.; Pelin, K.; Manzur, A. Y.; Mercuri, E.; Dubowitz, V.; Muntoni, F., Mild phenotype of nemaline myopathy with sleep hypoventilation due to a mutation in the skeletal muscle alpha-actin (ACTA1) gene. *Neuromuscular disorders : NMD* **2001,** 11, (1), 35-40.
96. Kao, J. C.; Liewluck, T.; Milone, M., A novel ACTA1 mutation causing progressive facioscapuloperoneal myopathy in an adult. *Journal of clinical neuroscience : official journal of the Neurosurgical Society of Australasia* **2018,** 53, 261-262.
97. Hoedemaekers, Y. M.; Caliskan, K.; Michels, M.; Frohn-Mulder, I.; van der Smagt, J. J.; Phefferkorn, J. E.; Wessels, M. W.; ten Cate, F. J.; Sijbrands, E. J.; Dooijes, D.; Majoor-Krakauer, D. F., The importance of genetic counseling, DNA diagnostics, and cardiologic family screening in left ventricular noncompaction cardiomyopathy. *Circulation. Cardiovascular genetics* **2010,** 3, (3), 232-9.
98. Olivotto, I.; Girolami, F.; Ackerman, M. J.; Nistri, S.; Bos, J. M.; Zachara, E.; Ommen, S. R.; Theis, J. L.; Vaubel, R. A.; Re, F.; Armentano, C.; Poggesi, C.; Torricelli, F.; Cecchi, F., Myofilament protein gene mutation screening and outcome of patients with hypertrophic cardiomyopathy. *Mayo Clinic proceedings* **2008,** 83, (6), 630-8.
99. Rodriguez-Serrano, M.; Domingo, D.; Igual, B.; Cano, A.; Medina, P.; Zorio, E., Familial left ventricular noncompaction associated with a novel mutation in the alpha-cardiac actin gene. *Revista espanola de cardiologia (English ed.)* **2014,** 67, (10), 857-9.

100. Powis, Z.; Farwell Hagman, K. D.; Speare, V.; Cain, T.; Blanco, K.; Mowlavi, L. S.; Mayerhofer, E. M.; Tilstra, D.; Vedder, T.; Hunter, J. M.; Tsang, M.; Gonzalez, L.; Vockley, G.; Tang, S., Exome sequencing in neonates: diagnostic rates, characteristics, and time to diagnosis. *Genetics in medicine : official journal of the American College of Medical Genetics* **2018,** 20, (11), 1468-1471.
101. Norrish, G.; Jager, J.; Field, E.; Quinn, E.; Fell, H.; Lord, E.; Cicerchia, M. N.; Ochoa, J. P.; Cervi, E.; Elliott, P. M.; Kaski, J. P., Yield of Clinical Screening for Hypertrophic Cardiomyopathy in Child First-Degree Relatives. *Circulation* **2019,** 140, (3), 184-192.
102. Sheikh, N.; Papadakis, M.; Wilson, M.; Malhotra, A.; Adamuz, C.; Homfray, T.; Monserrat, L.; Behr, E. R.; Sharma, S., Diagnostic Yield of Genetic Testing in Young Athletes With T-Wave Inversion. *Circulation* **2018,** 138, (12), 1184-1194.
103. Mogensen, J.; Klausen, I. C.; Pedersen, A. K.; Egeblad, H.; Bross, P.; Kruse, T. A.; Gregersen, N.; Hansen, P. S.; Baandrup, U.; Borglum, A. D., Alpha-cardiac actin is a novel disease gene in familial hypertrophic cardiomyopathy. *The Journal of clinical investigation* **1999,** 103, (10), R39-43.
104. Zenagui, R.; Lacourt, D.; Pegeot, H.; Yauy, K.; Juntas Morales, R.; Theze, C.; Rivier, F.; Cances, C.; Sole, G.; Renard, D.; Walther-Louvier, U.; Ferrer-Monasterio, X.; Espil, C.; Arne-Bes, M. C.; Cintas, P.; Uro-Coste, E.; Martin Negrier, M. L.; Rigau, V.; Bieth, E.; Goizet, C.; Claustres, M.; Koenig, M.; Cossee, M., A Reliable Targeted Next-Generation Sequencing Strategy for Diagnosis of Myopathies and Muscular Dystrophies, Especially for the Giant Titin and Nebulin Genes. *The Journal of molecular diagnostics : JMD* **2018,** 20, (4), 533-549.
105. Campens, L.; Callewaert, B.; Muino Mosquera, L.; Renard, M.; Symoens, S.; De Paepe, A.; Coucke, P.; De Backer, J., Gene panel sequencing in heritable thoracic aortic disorders and related entities - results of comprehensive testing in a cohort of 264 patients. *Orphanet journal of rare diseases* **2015,** 10, 9.
106. Dai, Y.; Wei, X.; Zhao, Y.; Ren, H.; Lan, Z.; Yang, Y.; Chen, L.; Cui, L., A comprehensive genetic diagnosis of Chinese muscular dystrophy and congenital myopathy patients by targeted next-generation sequencing. *Neuromuscular disorders : NMD* **2015,** 25, (8), 617-24.
107. Kaski, J. P.; Syrris, P.; Burch, M.; Tome-Esteban, M. T.; Fenton, M.; Christiansen, M.; Andersen, P. S.; Sebire, N.; Ashworth, M.; Deanfield, J. E.; McKenna, W. J.; Elliott, P. M., Idiopathic restrictive cardiomyopathy in children is caused by mutations in cardiac sarcomere protein genes. *Heart (British Cardiac Society)* **2008,** 94, (11), 1478-84.
108. Kaski, J. P.; Syrris, P.; Esteban, M. T.; Jenkins, S.; Pantazis, A.; Deanfield, J. E.; McKenna, W. J.; Elliott, P. M., Prevalence of sarcomere protein gene mutations in preadolescent children with hypertrophic cardiomyopathy. *Circulation. Cardiovascular genetics* **2009,** 2, (5), 436-41.
109. Olson, T. M.; Michels, V. V.; Thibodeau, S. N.; Tai, Y. S.; Keating, M. T., Actin mutations in dilated cardiomyopathy, a heritable form of heart failure. *Science (New York, N.Y.)* **1998,** 280, (5364), 750-2.
110. Maron, B. J.; Maron, M. S.; Semsarian, C., Double or compound sarcomere mutations in hypertrophic cardiomyopathy: a potential link to sudden death in the absence of conventional risk factors. *Heart rhythm* **2012,** 9, (1), 57-63.
111. Wu, L.; Brady, L.; Shoffner, J.; Tarnopolsky, M. A., Next-Generation Sequencing to Diagnose Muscular Dystrophy, Rhabdomyolysis, and HyperCKemia. *The Canadian journal of neurological sciences. Le journal canadien des sciences neurologiques* **2018,** 45, (3), 262-268.
112. Kajino, S.; Ishihara, K.; Goto, K.; Ishigaki, K.; Noguchi, S.; Nonaka, I.; Osawa, M.; Nishino, I.; Hayashi, Y. K., Congenital fiber type disproportion myopathy caused by LMNA mutations. *Journal of the neurological sciences* **2014,** 340, (1-2), 94-8.
113. Ware, S. M.; Shikany, A.; Landis, B. J.; James, J. F.; Hinton, R. B., Twins with progressive thoracic aortic aneurysm, recurrent dissection and ACTA2 mutation. *Pediatrics* **2014,** 134, (4), e1218-23.

114. Tian, T.; Wang, J.; Wang, H.; Sun, K.; Wang, Y.; Jia, L.; Zou, Y.; Hui, R.; Zhou, X.; Song, L., A low prevalence of sarcomeric gene variants in a Chinese cohort with left ventricular non-compaction. *Heart and vessels* **2015,** 30, (2), 258-64.
115. Laing, N. G.; Clarke, N. F.; Dye, D. E.; Liyanage, K.; Walker, K. R.; Kobayashi, Y.; Shimakawa, S.; Hagiwara, T.; Ouvrier, R.; Sparrow, J. C.; Nishino, I.; North, K. N.; Nonaka, I., Actin mutations are one cause of congenital fibre type disproportion. *Annals of neurology* **2004,** 56, (5), 689-94.
116. D'Amico, A.; Graziano, C.; Pacileo, G.; Petrini, S.; Nowak, K. J.; Boldrini, R.; Jacques, A.; Feng, J. J.; Porfirio, B.; Sewry, C. A.; Santorelli, F. M.; Limongelli, G.; Bertini, E.; Laing, N.; Marston, S. B., Fatal hypertrophic cardiomyopathy and nemaline myopathy associated with ACTA1 K336E mutation. *Neuromuscular disorders : NMD* **2006,** 16, (9-10), 548-52.
117. Huang, K.; Luo, Y. E.; Li, Q. X.; Duan, H. Q.; Bi, F. F.; Yang, H.; Luo, Y. B., [Clinical, pathological and genetic studies of two cases of childhood-onset nemaline myopathy]. *Zhongguo dang dai er ke za zhi = Chinese journal of contemporary pediatrics* **2018,** 20, (10), 804-808.
118. Sewry, C. A.; Holton, J. L.; Dick, D. J.; Muntoni, F.; Hanna, M. G., Zebra body myopathy is caused by a mutation in the skeletal muscle actin gene (ACTA1). *Neuromuscular disorders : NMD* **2015,** 25, (5), 388-91.
119. Gatayama, R.; Ueno, K.; Nakamura, H.; Yanagi, S.; Ueda, H.; Yamagishi, H.; Yasui, S., Nemaline myopathy with dilated cardiomyopathy in childhood. *Pediatrics* **2013,** 131, (6), e1986-90.
120. Fromer, M.; Pocklington, A. J.; Kavanagh, D. H.; Williams, H. J.; Dwyer, S.; Gormley, P.; Georgieva, L.; Rees, E.; Palta, P.; Ruderfer, D. M.; Carrera, N.; Humphreys, I.; Johnson, J. S.; Roussos, P.; Barker, D. D.; Banks, E.; Milanova, V.; Grant, S. G.; Hannon, E.; Rose, S. A.; Chambert, K.; Mahajan, M.; Scolnick, E. M.; Moran, J. L.; Kirov, G.; Palotie, A.; McCarroll, S. A.; Holmans, P.; Sklar, P.; Owen, M. J.; Purcell, S. M.; O'Donovan, M. C., De novo mutations in schizophrenia implicate synaptic networks. *Nature* **2014,** 506, (7487), 179-84.
121. Haas, J.; Frese, K. S.; Peil, B.; Kloos, W.; Keller, A.; Nietsch, R.; Feng, Z.; Muller, S.; Kayvanpour, E.; Vogel, B.; Sedaghat-Hamedani, F.; Lim, W. K.; Zhao, X.; Fradkin, D.; Kohler, D.; Fischer, S.; Franke, J.; Marquart, S.; Barb, I.; Li, D. T.; Amr, A.; Ehlermann, P.; Mereles, D.; Weis, T.; Hassel, S.; Kremer, A.; King, V.; Wirsz, E.; Isnard, R.; Komajda, M.; Serio, A.; Grasso, M.; Syrris, P.; Wicks, E.; Plagnol, V.; Lopes, L.; Gadgaard, T.; Eiskjaer, H.; Jorgensen, M.; Garcia-Giustiniani, D.; Ortiz-Genga, M.; Crespo-Leiro, M. G.; Deprez, R. H.; Christiaans, I.; van Rijsingen, I. A.; Wilde, A. A.; Waldenstrom, A.; Bolognesi, M.; Bellazzi, R.; Morner, S.; Bermejo, J. L.; Monserrat, L.; Villard, E.; Mogensen, J.; Pinto, Y. M.; Charron, P.; Elliott, P.; Arbustini, E.; Katus, H. A.; Meder, B., Atlas of the clinical genetics of human dilated cardiomyopathy. *European heart journal* **2015,** 36, (18), 1123-35a.
122. Bouldin, A. A.; Parisi, M. A.; Laing, N.; Patterson, K.; Gospe, S. M., Jr., Variable presentation of nemaline myopathy: novel mutation of alpha actin gene. *Muscle & nerve* **2007,** 35, (2), 254-8.
123. Wallefeld, W.; Krause, S.; Nowak, K. J.; Dye, D.; Horvath, R.; Molnar, Z.; Szabo, M.; Hashimoto, K.; Reina, C.; De Carlos, J.; Rosell, J.; Cabello, A.; Navarro, C.; Nishino, I.; Lochmuller, H.; Laing, N. G., Severe nemaline myopathy caused by mutations of the stop codon of the skeletal muscle alpha actin gene (ACTA1). *Neuromuscular disorders : NMD* **2006,** 16, (9-10), 541-7.
124. Di Donato, N.; Kuechler, A.; Vergano, S.; Heinritz, W.; Bodurtha, J.; Merchant, S. R.; Breningstall, G.; Ladda, R.; Sell, S.; Altmuller, J.; Bogershausen, N.; Timms, A. E.; Hackmann, K.; Schrock, E.; Collins, S.; Olds, C.; Rump, A.; Dobyns, W. B., Update on the ACTG1-associated Baraitser-Winter cerebrofrontofacial syndrome. *American journal of medical genetics. Part A* **2016,** 170, (10), 2644-51.
125. Riviere, J. B.; van Bon, B. W.; Hoischen, A.; Kholmanskikh, S. S.; O'Roak, B. J.; Gilissen, C.; Gijsen, S.; Sullivan, C. T.; Christian, S. L.; Abdul-Rahman, O. A.; Atkin, J. F.; Chassaing, N.; Drouin-Garraud, V.; Fry, A. E.; Fryns, J. P.; Gripp, K. W.; Kempers, M.; Kleefstra, T.; Mancini,

G. M.; Nowaczyk, M. J.; van Ravenswaaij-Arts, C. M.; Roscioli, T.; Marble, M.; Rosenfeld, J. A.; Siu, V. M.; de Vries, B. B.; Shendure, J.; Verloes, A.; Veltman, J. A.; Brunner, H. G.; Ross, M. E.; Pilz, D. T.; Dobyns, W. B., De novo mutations in the actin genes ACTB and ACTG1 cause Baraitser-Winter syndrome. *Nature genetics* **2012,** 44, (4), 440-4, s1-2.

126. Verloes, A.; Di Donato, N.; Masliah-Planchon, J.; Jongmans, M.; Abdul-Raman, O. A.; Albrecht, B.; Allanson, J.; Brunner, H.; Bertola, D.; Chassaing, N.; David, A.; Devriendt, K.; Eftekhari, P.; Drouin-Garraud, V.; Faravelli, F.; Faivre, L.; Giuliano, F.; Guion Almeida, L.; Juncos, J.; Kempers, M.; Eker, H. K.; Lacombe, D.; Lin, A.; Mancini, G.; Melis, D.; Lourenco, C. M.; Siu, V. M.; Morin, G.; Nezarati, M.; Nowaczyk, M. J.; Ramer, J. C.; Osimani, S.; Philip, N.; Pierpont, M. E.; Procaccio, V.; Roseli, Z. S.; Rossi, M.; Rusu, C.; Sznajer, Y.; Templin, L.; Uliana, V.; Klaus, M.; Van Bon, B.; Van Ravenswaaij, C.; Wainer, B.; Fry, A. E.; Rump, A.; Hoischen, A.; Drunat, S.; Riviere, J. B.; Dobyns, W. B.; Pilz, D. T., Baraitser-Winter cerebrofrontofacial syndrome: delineation of the spectrum in 42 cases. *European journal of human genetics : EJHG* **2015,** 23, (3), 292-301.

127. Wang, H.; Guan, J.; Lan, L.; Yu, L.; Xie, L.; Liu, X.; Yang, J.; Zhao, C.; Wang, D.; Wang, Q., A novel de novo mutation of ACTG1 in two sporadic non-syndromic hearing loss cases. *Science China. Life sciences* **2018,** 61, (6), 729-732.

128. Posey, J. E.; Harel, T.; Liu, P.; Rosenfeld, J. A.; James, R. A.; Coban Akdemir, Z. H.; Walkiewicz, M.; Bi, W.; Xiao, R.; Ding, Y.; Xia, F.; Beaudet, A. L.; Muzny, D. M.; Gibbs, R. A.; Boerwinkle, E.; Eng, C. M.; Sutton, V. R.; Shaw, C. A.; Plon, S. E.; Yang, Y.; Lupski, J. R., Resolution of Disease Phenotypes Resulting from Multilocus Genomic Variation. *The New England journal of medicine* **2017,** 376, (1), 21-31.

129. Yates, T. M.; Turner, C. L.; Firth, H. V.; Berg, J.; Pilz, D. T., Baraitser-Winter cerebrofrontofacial syndrome. *Clinical genetics* **2017,** 92, (1), 3-9.

130. Miyagawa, M.; Nishio, S. Y.; Ichinose, A.; Iwasaki, S.; Murata, T.; Kitajiri, S.; Usami, S., Mutational spectrum and clinical features of patients with ACTG1 mutations identified by massively parallel DNA sequencing. *The Annals of otology, rhinology, and laryngology* **2015,** 124 Suppl 1, 84s-93s.

131. de Heer, A. M.; Huygen, P. L.; Collin, R. W.; Oostrik, J.; Kremer, H.; Cremers, C. W., Audiometric and vestibular features in a second Dutch DFNA20/26 family with a novel mutation in ACTG1. *The Annals of otology, rhinology, and laryngology* **2009,** 118, (5), 382-90.

132. Kemerley, A.; Sloan, C.; Pfeifer, W.; Smith, R.; Drack, A., A novel mutation in ACTG1 causing Baraitser-Winter syndrome with extremely variable expressivity in three generations. *Ophthalmic genetics* **2017,** 38, (2), 152-156.

133. Morgan, A.; Lenarduzzi, S.; Cappellani, S.; Pecile, V.; Morgutti, M.; Orzan, E.; Ghiselli, S.; Ambrosetti, U.; Brumat, M.; Gajendrarao, P.; La Bianca, M.; Faletra, F.; Grosso, E.; Sirchia, F.; Sensi, A.; Graziano, C.; Seri, M.; Gasparini, P.; Girotto, G., Genomic Studies in a Large Cohort of Hearing Impaired Italian Patients Revealed Several New Alleles, a Rare Case of Uniparental Disomy (UPD) and the Importance to Search for Copy Number Variations. *Frontiers in genetics* **2018,** 9, 681.

134. Sandestig, A.; Green, A.; Jonasson, J.; Vogt, H.; Wahlstrom, J.; Pepler, A.; Ellnebo, K.; Biskup, S.; Stefanova, M., Could Dissimilar Phenotypic Effects of ACTB Missense Mutations Reflect the Actin Conformational Change? Two Novel Mutations and Literature Review. *Molecular syndromology* **2019,** 9, (5), 259-265.

135. Rainger, J.; Williamson, K. A.; Soares, D. C.; Truch, J.; Kurian, D.; Gillessen-Kaesbach, G.; Seawright, A.; Prendergast, J.; Halachev, M.; Wheeler, A.; McTeir, L.; Gill, A. C.; van Heyningen, V.; Davey, M. G.; FitzPatrick, D. R., A recurrent de novo mutation in ACTG1 causes isolated ocular coloboma. *Human mutation* **2017,** 38, (8), 942-946.

136. Maddirevula, S.; Alsahli, S.; Alhabeeb, L.; Patel, N.; Alzahrani, F.; Shamseldin, H. E.; Anazi, S.; Ewida, N.; Alsaif, H. S.; Mohamed, J. Y.; Alazami, A. M.; Ibrahim, N.; Abdulwahab, F.; Hashem, M.; Abouelhoda, M.; Monies, D.; Al Tassan, N.; Alshammari, M.; Alsagheir, A.;

Seidahmed, M. Z.; Sogati, S.; Aglan, M. S.; Hamad, M. H.; Salih, M. A.; Hamed, A. A.; Alhashmi, N.; Nabil, A.; Alfadli, F.; Abdel-Salam, G. M. H.; Alkuraya, H.; Peitee, W. O.; Keng, W. T.; Qasem, A.; Mushiba, A. M.; Zaki, M. S.; Fassad, M. R.; Alfadhel, M.; Alexander, S.; Sabr, Y.; Temtamy, S.; Ekbote, A. V.; Ismail, S.; Hosny, G. A.; Otaify, G. A.; Amr, K.; Al Tala, S.; Khan, A. O.; Rizk, T.; Alaqeel, A.; Alsiddiky, A.; Singh, A.; Kapoor, S.; Alhashem, A.; Faqeih, E.; Shaheen, R.; Alkuraya, F. S., Expanding the phenome and variome of skeletal dysplasia. *Genetics in medicine : official journal of the American College of Medical Genetics* **2018,** 20, (12), 1609-1616.
137. Di Donato, N.; Rump, A.; Koenig, R.; Der Kaloustian, V. M.; Halal, F.; Sonntag, K.; Krause, C.; Hackmann, K.; Hahn, G.; Schrock, E.; Verloes, A., Severe forms of Baraitser-Winter syndrome are caused by ACTB mutations rather than ACTG1 mutations. *European journal of human genetics : EJHG* **2014,** 22, (2), 179-83.
138. Poirier, K.; Martinovic, J.; Laquerriere, A.; Cavallin, M.; Fallet-Bianco, C.; Desguerre, I.; Valence, S.; Grande-Goburghun, J.; Francannet, C.; Deleuze, J. F.; Boland, A.; Chelly, J.; Bahi-Buisson, N., Rare ACTG1 variants in fetal microlissencephaly. *European journal of medical genetics* **2015,** 58, (8), 416-8.
139. Sloan-Heggen, C. M.; Bierer, A. O.; Shearer, A. E.; Kolbe, D. L.; Nishimura, C. J.; Frees, K. L.; Ephraim, S. S.; Shibata, S. B.; Booth, K. T.; Campbell, C. A.; Ranum, P. T.; Weaver, A. E.; Black-Ziegelbein, E. A.; Wang, D.; Azaiez, H.; Smith, R. J. H., Comprehensive genetic testing in the clinical evaluation of 1119 patients with hearing loss. *Human genetics* **2016,** 135, (4), 441-450.
140. Zhu, M.; Yang, T.; Wei, S.; DeWan, A. T.; Morell, R. J.; Elfenbein, J. L.; Fisher, R. A.; Leal, S. M.; Smith, R. J.; Friderici, K. H., Mutations in the gamma-actin gene (ACTG1) are associated with dominant progressive deafness (DFNA20/26). *American journal of human genetics* **2003,** 73, (5), 1082-91.
141. Popp, B.; Ekici, A. B.; Thiel, C. T.; Hoyer, J.; Wiesener, A.; Kraus, C.; Reis, A.; Zweier, C., Exome Pool-Seq in neurodevelopmental disorders. *European journal of human genetics : EJHG* **2017,** 25, (12), 1364-1376.
142. Johnston, J. J.; Wen, K. K.; Keppler-Noreuil, K.; McKane, M.; Maiers, J. L.; Greiner, A.; Sapp, J. C.; Demali, K. A.; Rubenstein, P. A.; Biesecker, L. G., Functional analysis of a de novo ACTB mutation in a patient with atypical Baraitser-Winter syndrome. *Human mutation* **2013,** 34, (9), 1242-9.
143. Morin, M.; Bryan, K. E.; Mayo-Merino, F.; Goodyear, R.; Mencia, A.; Modamio-Hoybjor, S.; del Castillo, I.; Cabalka, J. M.; Richardson, G.; Moreno, F.; Rubenstein, P. A.; Moreno-Pelayo, M. A., In vivo and in vitro effects of two novel gamma-actin (ACTG1) mutations that cause DFNA20/26 hearing impairment. *Human molecular genetics* **2009,** 18, (16), 3075-89.
144. Liu, P.; Li, H.; Ren, X.; Mao, H.; Zhu, Q.; Zhu, Z.; Yang, R.; Yuan, W.; Liu, J.; Wang, Q.; Liu, M., Novel ACTG1 mutation causing autosomal dominant non-syndromic hearing impairment in a Chinese family. *Journal of genetics and genomics = Yi chuan xue bao* **2008,** 35, (9), 553-8.
145. Cabanillas, R.; Dineiro, M.; Cifuentes, G. A.; Castillo, D.; Pruneda, P. C.; Alvarez, R.; Sanchez-Duran, N.; Capin, R.; Plasencia, A.; Viejo-Diaz, M.; Garcia-Gonzalez, N.; Hernando, I.; Llorente, J. L.; Reparaz-Andrade, A.; Torreira-Banzas, C.; Rosell, J.; Govea, N.; Gomez-Martinez, J. R.; Nunez-Batalla, F.; Garrote, J. A.; Mazon-Gutierrez, A.; Costales, M.; Isidoro-Garcia, M.; Garcia-Berrocal, B.; Ordonez, G. R.; Cadinanos, J., Comprehensive genomic diagnosis of non-syndromic and syndromic hereditary hearing loss in Spanish patients. *BMC medical genomics* **2018,** 11, (1), 58.
146. Sun, Y.; Shen, X.; Li, Q.; Kong, Q., Child with cerebral malformations and epilepsy. *The International journal of neuroscience* **2018,** 128, (9), 881-885.

147. Miyagawa, M.; Nishio, S. Y.; Ikeda, T.; Fukushima, K.; Usami, S., Massively parallel DNA sequencing successfully identifies new causative mutations in deafness genes in patients with cochlear implantation and EAS. *PloS one* **2013,** 8, (10), e75793.
148. Longoni, M.; High, F. A.; Qi, H.; Joy, M. P.; Hila, R.; Coletti, C. M.; Wynn, J.; Loscertales, M.; Shan, L.; Bult, C. J.; Wilson, J. M.; Shen, Y.; Chung, W. K.; Donahoe, P. K., Genome-wide enrichment of damaging de novo variants in patients with isolated and complex congenital diaphragmatic hernia. *Human genetics* **2017,** 136, (6), 679-691.
149. Helbig, K. L.; Farwell Hagman, K. D.; Shinde, D. N.; Mroske, C.; Powis, Z.; Li, S.; Tang, S.; Helbig, I., Diagnostic exome sequencing provides a molecular diagnosis for a significant proportion of patients with epilepsy. *Genetics in medicine : official journal of the American College of Medical Genetics* **2016,** 18, (9), 898-905.
150. Procaccio, V.; Salazar, G.; Ono, S.; Styers, M. L.; Gearing, M.; Davila, A.; Jimenez, R.; Juncos, J.; Gutekunst, C. A.; Meroni, G.; Fontanella, B.; Sontag, E.; Sontag, J. M.; Faundez, V.; Wainer, B. H., A mutation of beta -actin that alters depolymerization dynamics is associated with autosomal dominant developmental malformations, deafness, and dystonia. *American journal of human genetics* **2006,** 78, (6), 947-60.
151. Baek, J. I.; Oh, S. K.; Kim, D. B.; Choi, S. Y.; Kim, U. K.; Lee, K. Y.; Lee, S. H., Targeted massive parallel sequencing: the effective detection of novel causative mutations associated with hearing loss in small families. *Orphanet journal of rare diseases* **2012,** 7, 60.
152. Retterer, K.; Juusola, J.; Cho, M. T.; Vitazka, P.; Millan, F.; Gibellini, F.; Vertino-Bell, A.; Smaoui, N.; Neidich, J.; Monaghan, K. G.; McKnight, D.; Bai, R.; Suchy, S.; Friedman, B.; Tahiliani, J.; Pineda-Alvarez, D.; Richard, G.; Brandt, T.; Haverfield, E.; Chung, W. K.; Bale, S., Clinical application of whole-exome sequencing across clinical indications. *Genetics in medicine : official journal of the American College of Medical Genetics* **2016,** 18, (7), 696-704.
153. Vontell, R.; Supramaniam, V. G.; Davidson, A.; Thornton, C.; Marnerides, A.; Holder-Espinasse, M.; Lillis, S.; Yau, S.; Jansson, M.; Hagberg, H. E.; Rutherford, M. A., Post-mortem Characterisation of a Case With an ACTG1 Variant, Agenesis of the Corpus Callosum and Neuronal Heterotopia. *Frontiers in physiology* **2019,** 10, 623.
154. Boissel, S.; Fallet-Bianco, C.; Chitayat, D.; Kremer, V.; Nassif, C.; Rypens, F.; Delrue, M. A.; Dal Soglio, D.; Oligny, L. L.; Patey, N.; Flori, E.; Cloutier, M.; Dyment, D.; Campeau, P.; Karalis, A.; Nizard, S.; Fraser, W. D.; Audibert, F.; Lemyre, E.; Rouleau, G. A.; Hamdan, F. F.; Kibar, Z.; Michaud, J. L., Genomic study of severe fetal anomalies and discovery of GREB1L mutations in renal agenesis. *Genetics in medicine : official journal of the American College of Medical Genetics* **2018,** 20, (7), 745-753.
155. Diets, I. J.; Waanders, E.; Ligtenberg, M. J.; van Bladel, D. A. G.; Kamping, E. J.; Hoogerbrugge, P. M.; Hopman, S.; Olderode-Berends, M. J.; Gerkes, E. H.; Koolen, D. A.; Marcelis, C.; Santen, G. W.; van Belzen, M. J.; Mordaunt, D.; McGregor, L.; Thompson, E.; Kattamis, A.; Pastorczak, A.; Mlynarski, W.; Ilencikova, D.; van Silfhout, A. V.; Gardeitchik, T.; de Bont, E. S.; Loeffen, J.; Wagner, A.; Mensenkamp, A. R.; Kuiper, R. P.; Hoogerbrugge, N.; Jongmans, M. C., High Yield of Pathogenic Germline Mutations Causative or Likely Causative of the Cancer Phenotype in Selected Children with Cancer. *Clinical cancer research : an official journal of the American Association for Cancer Research* **2018,** 24, (7), 1594-1603.
156. Thiffault, I.; Farrow, E.; Zellmer, L.; Berrios, C.; Miller, N.; Gibson, M.; Caylor, R.; Jenkins, J.; Faller, D.; Soden, S.; Saunders, C., Clinical genome sequencing in an unbiased pediatric cohort. *Genetics in medicine : official journal of the American College of Medical Genetics* **2019,** 21, (2), 303-310.
157. Yuan, Y.; Gao, X.; Huang, B.; Lu, J.; Wang, G.; Lin, X.; Qu, Y.; Dai, P., Phenotypic Heterogeneity in a DFNA20/26 family segregating a novel ACTG1 mutation. *BMC genetics* **2016,** 17, 33.

158. Okamoto, N.; Miya, F.; Tsunoda, T.; Kato, M.; Saitoh, S.; Yamasaki, M.; Shimizu, A.; Torii, C.; Kanemura, Y.; Kosaki, K., Targeted next-generation sequencing in the diagnosis of neurodevelopmental disorders. *Clinical genetics* **2015,** 88, (3), 288-92.
159. Jin, S. C.; Homsy, J.; Zaidi, S.; Lu, Q.; Morton, S.; DePalma, S. R.; Zeng, X.; Qi, H.; Chang, W.; Sierant, M. C.; Hung, W. C.; Haider, S.; Zhang, J.; Knight, J.; Bjornson, R. D.; Castaldi, C.; Tikhonoa, I. R.; Bilguvar, K.; Mane, S. M.; Sanders, S. J.; Mital, S.; Russell, M. W.; Gaynor, J. W.; Deanfield, J.; Giardini, A.; Porter, G. A., Jr.; Srivastava, D.; Lo, C. W.; Shen, Y.; Watkins, W. S.; Yandell, M.; Yost, H. J.; Tristani-Firouzi, M.; Newburger, J. W.; Roberts, A. E.; Kim, R.; Zhao, H.; Kaltman, J. R.; Goldmuntz, E.; Chung, W. K.; Seidman, J. G.; Gelb, B. D.; Seidman, C. E.; Lifton, R. P.; Brueckner, M., Contribution of rare inherited and de novo variants in 2,871 congenital heart disease probands. *Nature genetics* **2017,** 49, (11), 1593-1601.
160. Zazo Seco, C.; Wesdorp, M.; Feenstra, I.; Pfundt, R.; Hehir-Kwa, J. Y.; Lelieveld, S. H.; Castelein, S.; Gilissen, C.; de Wijs, I. J.; Admiraal, R. J.; Pennings, R. J.; Kunst, H. P.; van de Kamp, J. M.; Tamminga, S.; Houweling, A. C.; Plomp, A. S.; Maas, S. M.; de Koning Gans, P. A.; Kant, S. G.; de Geus, C. M.; Frints, S. G.; Vanhoutte, E. K.; van Dooren, M. F.; van den Boogaard, M. H.; Scheffer, H.; Nelen, M.; Kremer, H.; Hoefsloot, L.; Schraders, M.; Yntema, H. G., The diagnostic yield of whole-exome sequencing targeting a gene panel for hearing impairment in The Netherlands. *European journal of human genetics : EJHG* **2017,** 25, (3), 308-314.
161. Weitensteiner, V.; Zhang, R.; Bungenberg, J.; Marks, M.; Gehlen, J.; Ralser, D. J.; Hilger, A. C.; Sharma, A.; Schumacher, J.; Gembruch, U.; Merz, W. M.; Becker, A.; Altmuller, J.; Thiele, H.; Herrmann, B. G.; Odermatt, B.; Ludwig, M.; Reutter, H., Exome sequencing in syndromic brain malformations identifies novel mutations in ACTB, and SLC9A6, and suggests BAZ1A as a new candidate gene. *Birth defects research* **2018,** 110, (7), 587-597.
162. Mutai, H.; Suzuki, N.; Shimizu, A.; Torii, C.; Namba, K.; Morimoto, N.; Kudoh, J.; Kaga, K.; Kosaki, K.; Matsunaga, T., Diverse spectrum of rare deafness genes underlies early-childhood hearing loss in Japanese patients: a cross-sectional, multi-center next-generation sequencing study. *Orphanet journal of rare diseases* **2013,** 8, 172.
163. van Wijk, E.; Krieger, E.; Kemperman, M. H.; De Leenheer, E. M.; Huygen, P. L.; Cremers, C. W.; Cremers, F. P.; Kremer, H., A mutation in the gamma actin 1 (ACTG1) gene causes autosomal dominant hearing loss (DFNA20/26). *Journal of medical genetics* **2003,** 40, (12), 879-84.
164. Miyagawa, M.; Naito, T.; Nishio, S. Y.; Kamatani, N.; Usami, S., Targeted exon sequencing successfully discovers rare causative genes and clarifies the molecular epidemiology of Japanese deafness patients. *PloS one* **2013,** 8, (8), e71381.
165. Park, G.; Gim, J.; Kim, A. R.; Han, K. H.; Kim, H. S.; Oh, S. H.; Park, T.; Park, W. Y.; Choi, B. Y., Multiphasic analysis of whole exome sequencing data identifies a novel mutation of ACTG1 in a nonsyndromic hearing loss family. *BMC genomics* **2013,** 14, 191.
166. Latham, S. L.; Ehmke, N.; Reinke, P. Y. A.; Taft, M. H.; Eicke, D.; Reindl, T.; Stenzel, W.; Lyons, M. J.; Friez, M. J.; Lee, J. A.; Hecker, R.; Fruhwald, M. C.; Becker, K.; Neuhann, T. M.; Horn, D.; Schrock, E.; Niehaus, I.; Sarnow, K.; Grutzmann, K.; Gawehn, L.; Klink, B.; Rump, A.; Chaponnier, C.; Figueiredo, C.; Knofler, R.; Manstein, D. J.; Di Donato, N., Variants in exons 5 and 6 of ACTB cause syndromic thrombocytopenia. *Nature communications* **2018,** 9, (1), 4250.
167. Wei, Q.; Zhu, H.; Qian, X.; Chen, Z.; Yao, J.; Lu, Y.; Cao, X.; Xing, G., Targeted genomic capture and massively parallel sequencing to identify novel variants causing Chinese hereditary hearing loss. *Journal of translational medicine* **2014,** 12, 311.
168. Vona, B.; Muller, T.; Nanda, I.; Neuner, C.; Hofrichter, M. A.; Schroder, J.; Bartsch, O.; Lassig, A.; Keilmann, A.; Schraven, S.; Kraus, F.; Shehata-Dieler, W.; Haaf, T., Targeted next-generation sequencing of deafness genes in hearing-impaired individuals uncovers

informative mutations. *Genetics in medicine : official journal of the American College of Medical Genetics* **2014,** 16, (12), 945-53.
169. Sakuma, N.; Moteki, H.; Takahashi, M.; Nishio, S. Y.; Arai, Y.; Yamashita, Y.; Oridate, N.; Usami, S., An effective screening strategy for deafness in combination with a next-generation sequencing platform: a consecutive analysis. *Journal of human genetics* **2016,** 61, (3), 253-61.
170. Nunoi, H.; Yamazaki, T.; Tsuchiya, H.; Kato, S.; Malech, H. L.; Matsuda, I.; Kanegasaki, S., A heterozygous mutation of beta-actin associated with neutrophil dysfunction and recurrent infection. *Proceedings of the National Academy of Sciences of the United States of America* **1999,** 96, (15), 8693-8.
171. Rendtorff, N. D.; Zhu, M.; Fagerheim, T.; Antal, T. L.; Jones, M.; Teslovich, T. M.; Gillanders, E. M.; Barmada, M.; Teig, E.; Trent, J. M.; Friderici, K. H.; Stephan, D. A.; Tranebjaerg, L., A novel missense mutation in ACTG1 causes dominant deafness in a Norwegian DFNA20/26 family, but ACTG1 mutations are not frequent among families with hereditary hearing impairment. *European journal of human genetics : EJHG* **2006,** 14, (10), 1097-105.
172. Cuvertino, S.; Stuart, H. M.; Chandler, K. E.; Roberts, N. A.; Armstrong, R.; Bernardini, L.; Bhaskar, S.; Callewaert, B.; Clayton-Smith, J.; Davalillo, C. H.; Deshpande, C.; Devriendt, K.; Digilio, M. C.; Dixit, A.; Edwards, M.; Friedman, J. M.; Gonzalez-Meneses, A.; Joss, S.; Kerr, B.; Lampe, A. K.; Langlois, S.; Lennon, R.; Loget, P.; Ma, D. Y. T.; McGowan, R.; Des Medt, M.; O'Sullivan, J.; Odent, S.; Parker, M. J.; Pebrel-Richard, C.; Petit, F.; Stark, Z.; Stockler-Ipsiroglu, S.; Tinschert, S.; Vasudevan, P.; Villa, O.; White, S. M.; Zahir, F. R.; Woolf, A. S.; Banka, S., ACTB Loss-of-Function Mutations Result in a Pleiotropic Developmental Disorder. *American journal of human genetics* **2017,** 101, (6), 1021-1033.
173. Matera, I.; Rusmini, M.; Guo, Y.; Lerone, M.; Li, J.; Zhang, J.; Di Duca, M.; Nozza, P.; Mosconi, M.; Pini Prato, A.; Martucciello, G.; Barabino, A.; Morandi, F.; De Giorgio, R.; Stanghellini, V.; Ravazzolo, R.; Devoto, M.; Hakonarson, H.; Ceccherini, I., Variants of the ACTG2 gene correlate with degree of severity and presence of megacystis in chronic intestinal pseudo-obstruction. *European journal of human genetics : EJHG* **2016,** 24, (8), 1211-5.
174. Wangler, M. F.; Gonzaga-Jauregui, C.; Gambin, T.; Penney, S.; Moss, T.; Chopra, A.; Probst, F. J.; Xia, F.; Yang, Y.; Werlin, S.; Eglite, I.; Kornejeva, L.; Bacino, C. A.; Baldridge, D.; Neul, J.; Lehman, E. L.; Larson, A.; Beuten, J.; Muzny, D. M.; Jhangiani, S.; Gibbs, R. A.; Lupski, J. R.; Beaudet, A., Heterozygous de novo and inherited mutations in the smooth muscle actin (ACTG2) gene underlie megacystis-microcolon-intestinal hypoperistalsis syndrome. *PLoS genetics* **2014,** 10, (3), e1004258.
175. Halim, D.; Hofstra, R. M.; Signorile, L.; Verdijk, R. M.; van der Werf, C. S.; Sribudiani, Y.; Brouwer, R. W.; van, I. W. F.; Dahl, N.; Verheij, J. B.; Baumann, C.; Kerner, J.; van Bever, Y.; Galjart, N.; Wijnen, R. M.; Tibboel, D.; Burns, A. J.; Muller, F.; Brooks, A. S.; Alves, M. M., ACTG2 variants impair actin polymerization in sporadic Megacystis Microcolon Intestinal Hypoperistalsis Syndrome. *Human molecular genetics* **2016,** 25, (3), 571-83.
176. Maluleke, T.; Mangray, H.; Arnold, M.; Moore, H. A.; Moore, S. W., Recurrent ACTG2 gene variation in African degenerative visceral leiomyopathy. *Pediatric surgery international* **2019,** 35, (4), 439-442.
177. Collins, R. R. J.; Barth, B.; Megison, S.; Pfeifer, C. M.; Rice, L. M.; Harris, S.; Timmons, C. F.; Rakheja, D., ACTG2-Associated Visceral Myopathy With Chronic Intestinal Pseudoobstruction, Intestinal Malrotation, Hypertrophic Pyloric Stenosis, Choledochal Cyst, and a Novel Missense Mutation. *International journal of surgical pathology* **2019,** 27, (1), 77-83.
178. Ravenscroft, G.; Pannell, S.; O'Grady, G.; Ong, R.; Ee, H. C.; Faiz, F.; Marns, L.; Goel, H.; Kumarasinghe, P.; Sollis, E.; Sivadorai, P.; Wilson, M.; Magoffin, A.; Nightingale, S.; Freckmann, M. L.; Kirk, E. P.; Sachdev, R.; Lemberg, D. A.; Delatycki, M. B.; Kamm, M. A.; Basnayake, C.; Lamont, P. J.; Amor, D. J.; Jones, K.; Schilperoort, J.; Davis, M. R.; Laing, N. G., Variants in ACTG2 underlie a substantial number of Australasian patients with primary

chronic intestinal pseudo-obstruction. *Neurogastroenterology and motility : the official journal of the European Gastrointestinal Motility Society* **2018,** 30, (9), e13371.
179. Lehtonen, H. J.; Sipponen, T.; Tojkander, S.; Karikoski, R.; Jarvinen, H.; Laing, N. G.; Lappalainen, P.; Aaltonen, L. A.; Tuupanen, S., Segregation of a missense variant in enteric smooth muscle actin gamma-2 with autosomal dominant familial visceral myopathy. *Gastroenterology* **2012,** 143, (6), 1482-1491.e3.
180. Thorson, W.; Diaz-Horta, O.; Foster, J., 2nd; Spiliopoulos, M.; Quintero, R.; Farooq, A.; Blanton, S.; Tekin, M., De novo ACTG2 mutations cause congenital distended bladder, microcolon, and intestinal hypoperistalsis. *Human genetics* **2014,** 133, (6), 737-42.
181. Korgali, E. U.; Yavuz, A.; Simsek, C. E. C.; Guney, C.; Kurtulgan, H. K.; Baser, B.; Atalar, M. H.; Ozer, H.; Egilmez, H. R., Megacystis Microcolon Intestinal Hypoperistalsis Syndrome in Which a Different De Novo Actg2 Gene Mutation was Detected: A Case Report. *Fetal and pediatric pathology* **2018,** 37, (2), 109-116.
182. Moreno, C. A.; Metze, K.; Lomazi, E. A.; Bertola, D. R.; Barbosa, R. H.; Cosentino, V.; Sobreira, N.; Cavalcanti, D. P., Visceral myopathy: Clinical and molecular survey of a cohort of seven new patients and state of the art of overlapping phenotypes. *American journal of medical genetics. Part A* **2016,** 170, (11), 2965-2974.
183. Stark, Z.; Tan, T. Y.; Chong, B.; Brett, G. R.; Yap, P.; Walsh, M.; Yeung, A.; Peters, H.; Mordaunt, D.; Cowie, S.; Amor, D. J.; Savarirayan, R.; McGillivray, G.; Downie, L.; Ekert, P. G.; Theda, C.; James, P. A.; Yaplito-Lee, J.; Ryan, M. M.; Leventer, R. J.; Creed, E.; Macciocca, I.; Bell, K. M.; Oshlack, A.; Sadedin, S.; Georgeson, P.; Anderson, C.; Thorne, N.; Melbourne Genomics Health, A.; Gaff, C.; White, S. M., A prospective evaluation of whole-exome sequencing as a first-tier molecular test in infants with suspected monogenic disorders. *Genetics in medicine : official journal of the American College of Medical Genetics* **2016,** 18, (11), 1090-1096.
184. Whittington, J. R.; Poole, A. T.; Dutta, E. H.; Munn, M. B., A Novel Mutation in ACTG2 Gene in Mother with Chronic Intestinal Pseudoobstruction and Fetus with Megacystis Microcolon Intestinal Hypoperistalsis Syndrome. *Case reports in genetics* **2017,** 2017, 9146507.
185. Monies, D.; Maddirevula, S.; Kurdi, W.; Alanazy, M. H.; Alkhalidi, H.; Al-Owain, M.; Sulaiman, R. A.; Faqeih, E.; Goljan, E.; Ibrahim, N.; Abdulwahab, F.; Hashem, M.; Abouelhoda, M.; Shaheen, R.; Arold, S. T.; Alkuraya, F. S., Autozygosity reveals recessive mutations and novel mechanisms in dominant genes: implications in variant interpretation. *Genetics in medicine : official journal of the American College of Medical Genetics* **2017,** 19, (10), 1144-1150.
186. Iglesias, A.; Anyane-Yeboa, K.; Wynn, J.; Wilson, A.; Truitt Cho, M.; Guzman, E.; Sisson, R.; Egan, C.; Chung, W. K., The usefulness of whole-exome sequencing in routine clinical practice. *Genetics in medicine : official journal of the American College of Medical Genetics* **2014,** 16, (12), 922-31.
187. Wolny, M.; Colegrave, M.; Colman, L.; White, E.; Knight, P. J.; Peckham, M., Cardiomyopathy mutations in the tail of beta-cardiac myosin modify the coiled-coil structure and affect integration into thick filaments in muscle sarcomeres in adult cardiomyocytes. *J Biol Chem* **2013,** 288, (44), 31952-62.
